# Supplementary material for: Comparative mRNA booster effectiveness against death or hospitalization with COVID-19 pneumonia across at-risk US Veteran populations
Source: Nat Commun. 2023 May 23;14:2976. doi: 10.1038/s41467-023-38503-8 (PMC10205032; doi:10.1038/s41467-023-38503-8)
Supplement: Supplementary file 1 — Supplementary Information [file 41467_2023_38503_MOESM1_ESM.pdf]

Comparative mRNA booster effectiveness against death or hospitalization with COVID-19 pneumonia across at-risk US Veteran populations

Subtitle: mRNA-1273 for high-risk populations

Supplementary Material to:

Comparative mRNA booster effectiveness against death or hospitalization with COVID-19 pneumonia across at-risk US Veteran populations

Subtitle: mRNA-1273 for high-risk populations

J. Daniel Kelly[1,2,3,4*], MD PhD
Samuel Leonard[1], MS
W. John Boscardin[3], PhD
Katherine J. Hoggatt[1,2], PhD
Emily N. Lum[1], MPH
Charles C. Austin[5], MDiv
Amy Byers[1], PhD
Phyllis C. Tien[1,2], MD
Peter C. Austin,[6] PhD
Dawn M. Bravata[5,7,8], MD
Salomeh Keyhani[1,2], MD

Authors contributed equally.

Affiliations:
1. San Francisco VA Medical Center, San Francisco, CA, USA;
2. Department of Medicine, University of California, San Francisco (UCSF), CA, USA;
3. Department of Epidemiology and Biostatistics, UCSF, CA, USA;
4. F.I. Proctor Foundation, UCSF, CA, USA;
5. Department of Veterans Affairs (VA) Health Services and Development (HSR&D) Center for Health Information and Communication (CHIC) and the Department of Medicine, Richard L. Roudebush VA Medical Center; Indianapolis, IN, USA; Veterans Affairs Medical Center, Indianapolis, IN, USA;
6. Institute for Clinical Evaluative Sciences, Toronto, Ontario, Canada;
7. Department of Medicine, Indiana University School of Medicine, Indianapolis, IN, USA;
8. Regenstrief Institute, Indianapolis, IN, USA.

Contents

COVID-19 pneumonia ascertainment using a combination text-processing facilitated chart review and ICD codes ……………………………………………………………………………..4

Supplementary Figure 1. Cohort Selection in a Study of U.S. Veterans Who Received Booster Vaccination for COVID-19………………………………………………………………………5

Variable definitions and covariates included in the propensity score model………………………7

Immunocompromised status defined based on receipt of three classes of drugs………………14

Supplementary Table 1: Expanded table of characteristics of the boosted cohort by vaccination series……………………………………………………………………………………………..15

Supplementary Table 2: Characteristics of cohort by outcome…………………………………..17

Supplementary Table 3: Interaction analyses of booster type and hospitalization with COVID-19 pneumonia or death by Delta vs Omicron variant eras…………………………………………19

Supplementary Tables 4-9: Propensity weighted samples of veterans, overall and by sub-group, who received mRNA-1273 x3 or BNT-162b2 x3 COVID-19 vaccines……………………..20

COVID-19 pneumonia ascertainment using a combination text-processing facilitated chart review and ICD codes

To develop a text processing algorithm for rapid chart review, we stratified hospitalizations by the ICD code J12.82 and the presence of the word pneumonia in the medical record. We searched the VHA medical record during the hospitalization period for the word "pneumonia" using a text processing tool available on the VHA Informatics and Computing Infrastructure. Using the text processing tool and the ICD coded J12.82, we stratified a sample of hospitalizations into 4 groups: A) those with a discharge diagnosis of J12.82 and the word pneumonia in their notes; B) those without a discharge diagnosis of J12.82 but with the word pneumonia in their notes; C) those with a discharge diagnosis of J12.82 but without the word pneumonia in their notes ;and D) those with neither the code J12.82 nor the word pneumonia in their notes.

**Algorithm to identify patients admitted with COVID pneumonia (Total Reviewed to Develop Algorithm N=594)**		
	Discharge diagnosis J12.82 present	Discharge diagnosis J12.82 absent
Discharge summary or another note contains the word "pneumonia"	A=222	B=202
No note contains "pneumonia"	C=3	D=167

A: There were 222 patients who were discharged with the ICD code J12.82 and had the word pneumonia in hospital notes or in the discharge summary. We reviewed a random sample of 25 cases. All 25 cases had documented COVID-19 pneumonia.

B: There were 202 patients who did not have a discharge diagnosis of J12.82 but had the word pneumonia in hospital notes or in the discharge summary. We reviewed all 202 cases. Among the 202 cases review, 34 were identified by the care team as having COVID-19 pneumonia.

C: There were 3 hospitalizations with a discharge diagnosis of J12.82 but no note containing the word pneumonia. We reviewed all 3 cases. Among the 3 cases, two cases were identified by the care team as having COVID-19 pneumonia.

D: There were 167 cases with neither the code J12.82 nor the word pneumonia in their notes. We reviewed a random sample of 50 charts in this category. No patient was identified as having COVID-19 pneumonia in this group.

This algorithm demonstrated that all cases randomly sampled in Box A (cases with both the diagnosis code and the word pneumonia in the chart) were positive COVID-19 pneumonia cases and in Box B (cases without the code J12.82 and without the word pneumonia in the chart) were not COVID-19 pneumonia cases. Therefore, we did not conduct any further chart review of cases identified in Boxes A and B. This algorithm further demonstrated the presence of inconsistences among cases identified in Boxes B and C. For example, some cases were COVID-19 pneumonia even though the discharge diagnosis of J12.82 was absent. Therefore, we decided to conduct chart review of all cases identified in these Boxes (B and C). This algorithm was then applied to all patients who were hospitalized in the follow-up period.

Supplementary Figure 1. Cohort Selection in a Study of U.S. Veterans Who Received Booster Vaccination for COVID-19[a]

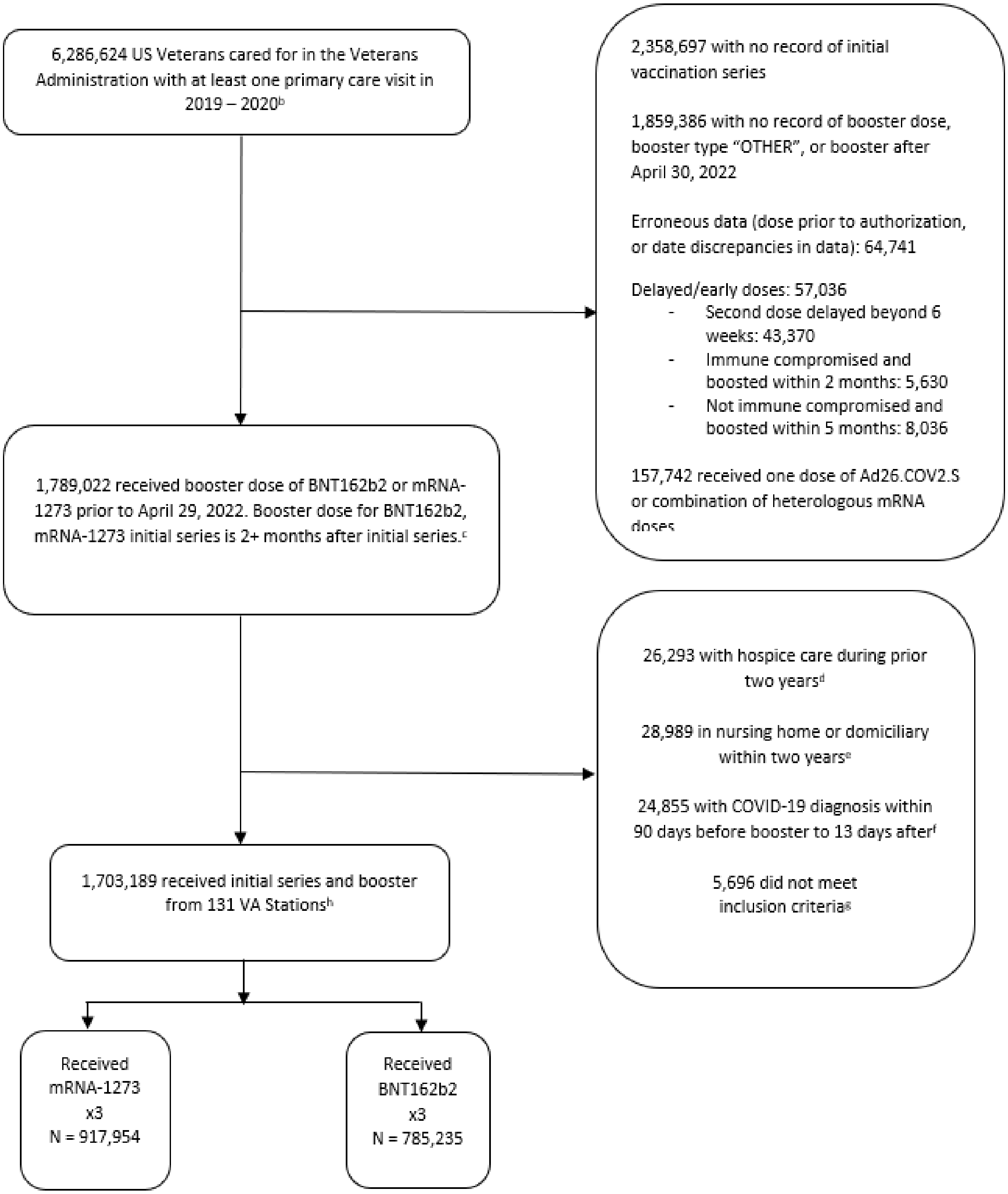

a. Eligibility based on first three doses.A small portion (~1%) of the overall cohort received a fourth dose before the end of follow-up because of their immunocompromised status.
b. Primary care visits defined as an outpatient visit with primary or secondary stop code:
   - 301 – general internal medicine
   - 318 – geriatrics
   - 319 – geriatrics

- 322 – women's health
- 323 – primary care medicine
- 338 – telephone primary care
- 348 – primary care shared appt
- 350 – geripact

c. Vaccine dates and types drawn from shared data resource ORDCOVID_Vaccine table
d. Hospice/palliative care is:
   - Receipt of ICD-10 diagnosis code Z51.5 (encounter for palliative care) in any context (in/outpatient, fee, etc.)
   - Any outpatient visit with primary or secondary stop code
     - o 351 (hospice care)
     - o 353 (palliative care)
   - Any single instance of a patient consult (from CDW Con_Consult table) where the service contains "hospice" or "palliative"
   - Any VA patient hospital stay with specialty containing "hospice" or "palliative"
   - Any fee basis hospital stay with "FeePurposeOfVisit" column containing "hospice" or "palliative" care.
e. Nursing home stays are:
   - Receipt of ICD-10 diagnosis code Y92.12x (nursing home as the place of occurrence of the external cause) in any context (in/outpatient, fee, etc.)
   - Any outpatient visit with primary or secondary stop code
     - o 119 – CNH follow up
     - o 121 – community res care
     - o 190 – adult day care
     - o 191 – community ADHC follow up
     - o 370 – GEC LTSS (geriatrics extended care – long term)
     - o 651 – state nursing home days
     - o 658 – state home adult day healthcare
   - Any VA inpatient stay with medical service "NHCU" or specialty "NH"
f. History of COVID is:
   - Any positive lab appearing in the shared data resource ORDCOVID_CaseLabChem table.
   - Any positive case in the shared data resource ORDCOVID_CaseDetail table.
   - Any single instance of ICD-10 diagnosis code U07.1 (COVID-19) or J12.89 (COVID-19 pneumonia) in any context (in/outpatient, VA/fee)
g. Patients were removed who had missing Care Assessment Need (CAN) score, had no age available, or had no VA priority level available
h. These 131 VA Stations represent all VA facilities.

Variable Definitions and Covariates Included in the Propensity Score Model

Covariates were age, sex, race, ethnicity, marital status, urban-rural residence, VHA facility variable (131VHA stations), body mass index (BMI), co-morbid conditions, history of prior SARS-CoV-2 infection, receipt of home-based primary care, and calendar time for vaccine series. Race and ethnicity have been associated with adverse COVID-19 outcomes in other populations; acquisition of race/ethnicity data in VHA occurred from patient or proxy self-report based on pre-specified categories defined in VHA Handbook 1601A.01.

Comorbid conditions associated with poor COVID-19 clinical outcomes in the literature (hospitalization and mortality) included hypertension, heart failure, ischemic heart disease, diabetes, stroke or transient ischemic attack, chronic obstructive pulmonary disease or bronchiectasis, cirrhosis, dementia, spinal cord injury , immunocompromised status in the past one year, any chronic kidney disease (CKD), severe CKD, dialysis, and cancer (solid organ, hematologic malignancies [lymphoma/leukemia], other). Behavioral risk factors were current smoking, alcohol use disorder, and any non-alcohol- or non-smoking-related substance use disorder. Social risk factors were housing problems (use of housing services in past year) and Veteran priority score (surrogate for income status). Reinfection was defined as two SARS-CoV-2 test results and/or COVID-19 diagnoses greater than 90 days apart.

Variable	Definition	
Hypertension	Two outpatient one inpatient code in past 2 years	I10, I11, I11.0, I11.9, I12.0, I12.9, I13, I13.0, I13.1, I13.10
Heart Failure	Two outpatient one inpatient code in past 2 years	I09.81, I11.0, I13.0, I13.2, I50.1, I50.20, I50.21, I50.22, I50.23, I50.30, I50.31, I50.32, I50.33, I50.40, I50.41, I50.42, I50.43, I50.814, I50.82, I50.83, I50.84, I50.89, I50.9
Ischemic Heart Disease	Two outpatient one inpatient code in past 2 years	I20.0, I20.8, I20.9, I21.01, I21.02, I21.09, I21.11, I21.19, I21.21, I21.29, I21.A9, I22.0, I22.1, I22.2, I22.8, I22.9, I23.0, I23.1, I23.2, I23.3, I23.4, I23.5, I23.6, I23.7, I23.8, I24.0, I24.1, I24.8, I24.9, I25.10, I25.110, I25.118, I25.119, I25.2, I25.3, I25.4, I25.5, I25.6, I25.7, I25.700, I25.708, I25.709, I25.710, I25.718, I25.719, I25.720, I25.728, I25.729, I25.730, I25.738, I25.739, I25.750, I25.758, I25.759, I25.760, I25.768, I25.769, I25.790, I25.798, I25.799, I25.810, I25.811, I25.812, I25.82, I25.83, I25.84, I25.89, I25.9, I51.2, Z98.61
Diabetes	Two outpatient one inpatient code in past 2 years	E08, E08.0, E08.00, E08.01, E08.1, E08.10, E08.11, E08.2, E08.21, E08.22, E08.29, E08.3, E08.31, E08.311, E08.319, E08.32, E08.321, E08.3211, E08.3212, E08.3213, E08.3219, E08.329, E08.3291, E08.3292, E08.3293, E08.3299, E08.33, E08.331, E08.3311, E08.3312, E08.3313, E08.3319, E08.339, E08.3391, E08.3392, E08.3393, E08.3399, E08.34, E08.341, E08.3411, E08.3412, E08.3413, E08.3419, E08.349, E08.3491, E08.3492, E08.3493, E08.3499, E08.35, E08.351, E08.3511, E08.3512, E08.3513, E08.3519, E08.352, E08.3521, E08.3522, E08.3523, E08.3529, E08.353, E08.3531, E08.3532, E08.3533, E08.3539, E08.354, E08.3541, E08.3542, E08.3543, E08.3549, E08.355, E08.3551, E08.3552, E08.3553, E08.3559, E08.359, E08.3591, E08.3592, E08.3593, E08.3599, E08.36, E08.37, E08.37X1, E08.37X2, E08.37X3, E08.37X9, E08.39, E08.4, E08.40, E08.41, E08.42, E08.43, E08.44, E08.49, E08.5, E08.51, E08.52, E08.59, E08.6, E08.61, E08.610, E08.618, E08.62, E08.620, E08.621, E08.622, E08.628, E08.63, E08.630, E08.638, E08.64, E08.641, E08.649, E08.65, E08.69, E08.8, E08.9, E09, E09.0, E09.00, E09.01, E09.1, E09.10, E09.11, E09.2, E09.21, E09.22, E09.29, E09.3, E09.31, E09.311, E09.319, E09.32, E09.321, E09.3211, E09.3212, E09.3213, E09.3219, E09.329, E09.3291, E09.3292, E09.3293, E09.3299, E09.33, E09.331, E09.3311, E09.3312, E09.3313, E09.3319,

Variable	Definition	
		E09.339, E09.3391, E09.3392, E09.3393, E09.3399, E09.34, E09.341, E09.3411, E09.3412, E09.3413, E09.3419, E09.349, E09.3491, E09.3492, E09.3493, E09.3499, E09.35, E09.351, E09.3511, E09.3512, E09.3513, E09.3519, E09.352, E09.3521, E09.3522, E09.3523, E09.3529, E09.353, E09.3531, E09.3532, E09.3533, E09.3539, E09.354, E09.3541, E09.3542, E09.3543, E09.3549, E09.355, E09.3551, E09.3552, E09.3553, E09.3559, E09.359, E09.3591, E09.3592, E09.3593, E09.3599, E09.36, E09.37, E09.37X1, E09.37X2, E09.37X3, E09.37X9, E09.39, E09.4, E09.40, E09.41, E09.42, E09.43, E09.44, E09.49, E09.5, E09.51, E09.52, E09.59, E09.6, E09.61, E09.610, E09.618, E09.62, E09.620, E09.621, E09.622, E09.628, E09.63, E09.630, E09.638, E09.64, E09.641, E09.649, E09.65, E09.69, E09.8, E09.9, E10, E10.1, E10.10, E10.11, E10.2, E10.21, E10.22, E10.29, E10.3, E10.31, E10.311, E10.319, E10.32, E10.321, E10.3211, E10.3212, E10.3213, E10.3219, E10.329, E10.3291, E10.3292, E10.3293, E10.3299, E10.33, E10.331, E10.3311, E10.3312, E10.3313, E10.3319, E10.339, E10.3391, E10.3392, E10.3393, E10.3399, E10.34, E10.341, E10.3411, E10.3412, E10.3413, E10.3419, E10.349, E10.3491, E10.3492, E10.3493, E10.3499, E10.35, E10.351, E10.3511, E10.3512, E10.3513, E10.3519, E10.352, E10.3521, E10.3522, E10.3523, E10.3529, E10.353, E10.3531, E10.3532, E10.3533, E10.3539, E10.354, E10.3541, E10.3542, E10.3543, E10.3549, E10.355, E10.3551, E10.3552, E10.3553, E10.3559, E10.359, E10.3591, E10.3592, E10.3593, E10.3599, E10.36, E10.37, E10.37X1, E10.37X2, E10.37X3, E10.37X9, E10.39, E10.4, E10.40, E10.41, E10.42, E10.43, E10.44, E10.49, E10.5, E10.51, E10.52, E10.59, E10.6, E10.61, E10.610, E10.618, E10.62, E10.620, E10.621, E10.622, E10.628, E10.63, E10.630, E10.638, E10.64, E10.641, E10.649, E10.65, E10.69, E10.8, E10.9, E11, E11.0, E11.00, E11.01, E11.1, E11.10, E11.11, E11.2, E11.21, E11.22, E11.29, E11.3, E11.31, E11.311, E11.319, E11.32, E11.321, E11.3211, E11.3212, E11.3213, E11.3219, E11.329, E11.3291, E11.3292, E11.3293, E11.3299, E11.33, E11.331, E11.3311, E11.3312, E11.3313, E11.3319, E11.339, E11.3391, E11.3392, E11.3393, E11.3399, E11.34, E11.341 E11.3411, E11.3412, E11.3413, E11.3419, E11.349, E11.3491, E11.3492, E11.3493, E11.3499, E11.35, E11.351, E11.3511, E11.3512, E11.3513, E11.3519, E11.352, E11.3521, E11.3522, E11.3523, E11.3529, E11.353, E11.3531, E11.3532, E11.3533, E11.3539, E11.354, E11.3541, E11.3542, E11.3543, E11.3549, E11.355, E11.3551, E11.3552, E11.3553, E11.3559, E11.359, E11.3591, E11.3592, E11.3593, E11.3599, E11.36, E11.37, E11.37X1, E11.37X2, E11.37X3, E11.37X9, E11.39, E11.4, E11.40, E11.41, E11.42, E11.43, E11.44, E11.49, E11.5, E11.51, E11.52, E11.59, E11.6, E11.61, E11.610, E11.618, E11.62, E11.620, E11.621, E11.622, E11.628, E11.63, E11.630, E11.638, E11.64, E11.641, E11.649, E11.65, E11.69, E11.8, E11.9, E13, E13.0, E13.00, E13.01, E13.1, E13.10, E13.11, E13.2, E13.21, E13.22, E13.29, E13.3, E13.31, E13.311, E13.319, E13.32, E13.321, E13.3211, E13.3212, E13.3213, E13.3219, E13.329, E13.3291, E13.3292, E13.3293, E13.3299, E13.33, E13.331, E13.3311, E13.3312, E13.3313, E13.3319, E13.339, E13.3391, E13.3392, E13.3393, E13.3399, E13.34, E13.341, E13.3411, E13.3412, E13.3413, E13.3419, E13.349, E13.3491, E13.3492, E13.3493, E13.3499, E13.35, E13.351, E13.3511, E13.3512, E13.3513, E13.3519, E13.352, E13.3521, E13.3522, E13.3523, E13.3529, E13.353, E13.3531, E13.3532, E13.3533, E13.3539, E13.354, E13.3541, E13.3542, E13.3543, E13.3549, E13.355,

Variable	Definition	
		E13.3551, E13.3552, E13.3553, E13.3559, E13.359, E13.3591, E13.3592, E13.3593, E13.3599, E13.36, E13.37, E13.37X1, E13.37X2, E13.37X3, E13.37X9, E13.39, E13.4, E13.40, E13.41, E13.42, E13.43, E13.44, E13.49, E13.5, E13.51, E13.52, E13.59, E13.6, E13.61, E13.610, E13.618, E13.62, E13.620, E13.621, E13.622, E13.628, E13.63, E13.630, E13.638, E13.64, E13.641, E13.649, E13.65, E13.69, E13.8, E13.9
Stroke or Transient Ischemic Attack (TIA)	Two outpatient one inpatient code in past 2 years	G45, G45.1, G45.3, G45.8, G45.9
COPD or Bronchiectasis	Two outpatient one inpatient code in past 2 years	J41, J41.0, J41.1, J41.8, J42, J43, J43.0, J43.1, J43.2, J43.8, J43.9, J44, J44.0, J44.1, J44.9
Cirrhosis	Two outpatient one inpatient code in past 2 years	B19.0, B19.11, B19.21, K65.2, K70.11, K70.2, K70.3, K70.30, K70.31, K70.41, K70.9, K71.11, K71.51, K71.7, K72.01, K72.11, K72.91, K74, K74.00, K74.01, K74.02, K74.1, K74.2, K74.3, K74.4, K74.5, K74.6, K74.60, K74.69, K76.6, K76.7, K76.81, I85, I85.0, I85.00, I85.01, I85.1, I85.10, I85.11, R18.8
Dialysis	Any single code in prior 6 months	VA algorithm, code available upon request
Chronic Kidney Disease	Most recent GFR prior to booster dose	Severe CKD was defined as GFR<30
Dementia	Two outpatient one inpatient code in past 2 years	F01, F01.5, F01.50, F01.51, F02, F02.8, F02.80, F02.81, F03, F03.9, F03.90, F03.91, F10.27, F10.97, F13.27, F13.97, F18.17, F18.27, F18.97, F19.17, F19.27, F19.97, G30, G30.0, G30.1, G30.8, G30.9, G31.0, G31.01, G31.09, G31.83
Immunocompromised	1) Receipt IV chemotherapy in the 90 days before vaccination completion<br>2) Receipt of immunosuppressant drugs<br>3) Receipt of antiretroviral drugs	See table below for complete list of drugs
Solid Organ Tumor	Head and Neck Cancer<br>Lung Cancer/respiratory tract<br>GI Tract Cancer<br>Other GI Cancer<br>Endocrine cancer<br>Genitourinary tract Cancer<br>Prostate cancer<br>CNS cancer<br>Bone/limb/connective tissue cancers<br>Other cancer	C00, C00.0, C00.1, C00.2, C00.3, C00.4, C00.5, C00.6, C00.8, C00.9, C43.0, C4A.0, C44.00, C44.01, C44.02, C44.09, C01, C02, C02.0, C02.1, C02.2, C02.3, C02.4, C02.8, C02.9, C03, C03.0, C03.1, C03.9, C04, C04.0, C04.1, C04.8, C04.9, C05, C05.0, C05.1, C05.2, C05.8, C05.9, C06, C06.0, C06.1, C06.2, C06.8, C06.80, C06.89, C06.9, C07, C08, C08.0, C08.1, C08.9, C09, C09.0, C09.1, C09.8, C09.9, C10, C10.0, C10.1, C10.2, C10.3, C10.4, C10.8, C10.9, C11, C11.0, C11.1, C11.2, C11.3, C11.8, C11.9, C12, C13, C13.0, C13.1, C13.2, C13.8, C13.9, C14, C14.0, C14.2, C14.8, C30, C30.0, C30.1, C31.0, C31.1, C31.2, C31.3, C31.8, C31.9, C32.0, C32.1, C32.2, C32.3, C32.8, C32.9, C33, C34.00, C34.01, C34.02, C34.10, C34.11, C34.12, C34.2, C34.30, C34.31, C34.32, C34.80, C34.81, C34.82, C34.90, C34.91, C34.92, C37, C38.0, C38.1, C38.2, C38.3, C38.4, C38.8, C39.0, C39.9, C31, C31.0, C31.1, C31.2, C31.3, C31.8, C31.9, C37, C69, C69.0, C69.00, C69.01, C69.02, C69.1, C69.10, C69.11, C69.12, C69.2, C69.20, C69.21, C69.22, C69.3, C69.30, C69.31, C69.32, C69.4, C69.40, C69.41, C69.42, C69.5, C69.50, C69.51, C69.52, C69.6, C69.60, C69.61, C69.62, C69.8, C69.80, C69.81, C69.82, C69.9, C69.90, C69.91, C69.92, C32, C32.0, C32.1, C32.2, C32.3, C32.8, C32.9, C33, C34, C34.0, C34.00, C34.01, C34.02, C34.1, C34.10, C34.11, C34.12, C34.2, C34.3, C34.30, C34.31, C34.32, C34.8, C34.80, C34.81, C34.82, C34.9, C34.90, C34.91, C34.92, C38, C38.0, C38.1, C38.2, C38.3, C38.4, C38.8, C39, C39.0, C39.9, C45, C45.0, C45.1, C45.2, C45.7, C45.9, C15, C15.3, C15.4, C15.5, C15.8, C15.9, C16, C16.0, C16.1, C16.2, C16.3, C16.4, C16.5, C16.6, C16.8, C16.9, C17, C17.0, C17.1, C17.2, C17.3, C17.8, C17.9, C18, C18.0, C18.1, C18.2, C18.3, C18.4, C18.5, C18.6, C18.7, C18.8, C18.9, C19, C20, C21, C21.0, C21.1, C21.2, C21.8, C22,

Variable	Definition	
		C22.0, C22.1, C22.2, C22.3, C22.4, C22.7, C22.8, C22.9, C23, C24, C24.0, C24.1, C24.8, C24.9, C25, C25.0, C25.1, C25.2, C25.3, C25.4, C25.7, C25.8, C25.9, C26, C26.0, C26.1, C46.4, C26.9, C48, C48.0, C48.1, C48.2, C48.8, C73, C74, C74.0, C74.00, C74.01, C74.02, C74.1, C74.10, C74.11, C74.12, C74.9, C74.90, C74.91, C74.92, C7A, C7A.0, C7A.00, C7A.01, C7A.010, C7A.011, C7A.012, C7A.019, C7A.02, C7A.020, C7A.021, C7A.022, C7A.023, C7A.024, C7A.025, C7A.026, C7A.029, C7A.09, C7A.090, C7A.091, C7A.092, C7A.093, C7A.094, C7A.095, C7A.096, C7A.098, C7A.1, C7A.8, C7B, C7B.0, C7B.00, C7B.01, C7B.02, C7B.03, C7B.04, C7B.09, C7B.1, C7B.8, C75, C75.0, C75.1, C75.2, C75.3, C75.4, C75.5, C75.8, C75.9, C62, C62.0, C62.00, C62.01, C62.02, C62.1, C62.10, C62.11, C62.12, C62.9, C62.90, C62.91, C62.92, C63, C63.0, C63.00, C63.01, C63.02, C63.1, C63.10, C63.11, C63.12, C63.2, C63.7, C63.8, C63.9, C64, C64.1, C64.2, C64.9, C65, C65.1, C65.2, C65.9, C67, C67.0, C67.1, C67.2, C67.3, C67.4, C67.5, C67.6, C67.7, C67.8, C67.9, C66, C66.1, C66.2, C66.9, C68, C68.0, C68.1, C68.8, C68.9, C61, C70, C70.0, C70.1, C70.9, C71, C71.0, C71.1, C71.2, C71.3, C71.4, C71.5, C71.6, C71.7, C71.8, C71.9, C72, C72.0, C72.1, C72.2, C72.20, C72.21, C72.22, C72.3, C72.30, C72.31, C72.32, C72.4, C72.40, C72.41, C72.42, C72.5, C72.50, C72.59, C72.9, C40, C40.0, C40.00, C40.01, C40.02, C40.1, C40.10, C40.11, C40.12, C40.2, C40.20, C40.21, C40.22, C40.3, C40.30, C40.31, C40.32, C40.8, C40.80, C40.81, C40.82, C40.9, C40.90, C40.91, C40.92, C41, C41.0, C41.1, C41.2, C41.3, C41.4, C41.9, C46, C46.0, C46.1, C46.2, C46.3, C46.4, C46.5, C46.50, C46.51, C46.52, C46.7, C46.9, C47, C47.0, C47.1, C47.10, C47.11, C47.12, C47.2, C47.20, C47.21, C47.22, C47.3, C47.4, C47.5, C47.6, C47.8, C47.9, C49, C49.0, C49.1, C49.10, C49.11, C49.12, C49.2, C49.20, C49.21, C49.22, C49.3, C49.4, C49.5, C49.6, C49.8, C49.9, C49.A, C49.A0, C49.A1, C49.A2, C49.A3, C49.A4, C49.A5, C49.A9, C76, C76.0, C76.1, C76.2, C76.3, C76.4, C76.40, C76.41, C76.42, C76.5, C76.50, C76.51, C76.52, C76.8, C79, C79.0, C79.00, C79.01, C79.02, C79.1, C79.10, C79.11, C79.19, C79.2, C79.3, C79.31, C79.32, C79.4, C79.40, C79.49, C79.5, C79.51, C79.52, C79.6, C79.60, C79.61, C79.62, C79.7, C79.70, C79.71, C79.72, C79.8, C79.81, C79.82, C79.89, C79.9, C78, C78.0, C78.00, C78.01, C78.02, C78.1, C78.2, C78.3, C78.30, C78.39, C78.4, C78.5, C78.6, C78.7, C78.8, C78.80, C78.89, C77, C77.0, C77.1, C77.2, C77.3, C77.4, C77.5, C77.8, C77.9, C80, C80.0, C80.1, C80.2
Hematologic malignancy	Hodgkin lymphoma<br>Follicular lymphoma<br>Non follicular lymphoma<br>TNK cell lymphoma<br>Other non-Hodgkin's lymphoma<br>Other specified TNK lymphoma<br>Immunoproliferative and b cell lymphoma<br>Multiple myeloma and plasma cell<br>Lymphoid leukemia<br>Myeloid leukemia<br>Monocytic leukemia<br>Leukemia & Specified Cell Type<br>Leukemia of unspecified cell<br>Unspecified malignant neoplasm	C81, C81.0, C81.00, C81.01, C81.02, C81.03, C81.04, C81.05, C81.06, C81.07, C81.08, C81.09, C81.1, C81.10, C81.11, C81.12, C81.13, C81.14, C81.15, C81.16, C81.17, C81.18, C81.19, C81.2, C81.20, C81.21, C81.22, C81.23, C81.24, C81.25, C81.26, C81.27, C81.28, C81.29, C81.3, C81.30, C81.31, C81.32, C81.33, C81.34, C81.35, C81.36, C81.37, C81.38, C81.39, C81.4, C81.40, C81.41, C81.42, C81.43, C81.44, C81.45, C81.46, C81.47, C81.48, C81.49, C81.7, C81.70, C81.71, C81.72, C81.73, C81.74, C81.75, C81.76, C81.77, C81.78, C81.79, C81.9, C81.90, C81.91, C81.92, C81.93, C81.94, C81.95, C81.96, C81.97, C81.98, C81.99, C82, C82.0, C82.00, C82.01, C82.02, C82.03, C82.04, C82.05, C82.06, C82.07, C82.08, C82.09,

Variable	Definition	
		C82.1, C82.10, C82.11, C82.12, C82.13, C82.14, C82.15, C82.16, C82.17, C82.18, C82.19, C82.2, C82.20, C82.21, C82.22, C82.23, C82.24, C82.25, C82.26, C82.27, C82.28, C82.29, C82.3, C82.30, C82.31, C82.32, C82.33, C82.34, C82.35, C82.36, C82.37, C82.38, C82.39, C82.4, C82.40, C82.41, C82.42, C82.43, C82.44, C82.45, C82.46, C82.47, C82.48, C82.49, C82.5, C82.50, C82.51, C82.52, C82.53, C82.54, C82.55, C82.56, C82.57, C82.58, C82.59, C82.6, C82.60, C82.61, C82.62, C82.63, C82.64, C82.65, C82.66, C82.67, C82.68, C82.69, C82.8, C82.80, C82.81, C82.82, C82.83, C82.84, C82.85, C82.86, C82.87, C82.88, C82.89, C82.9, C82.90, C82.91, C82.92, C82.93, C82.94, C82.95, C82.96, C82.97, C82.98, C82.99, C83, C83.0, C83.00, C83.01, C83.02, C83.03, C83.04, C83.05, C83.06, C83.07, C83.08, C83.09, C83.1, C83.10, C83.11, C83.12, C83.13, C83.14, C83.15, C83.16, C83.17, C83.18, C83.19, C83.3, C83.30, C83.31, C83.32, C83.33, C83.34, C83.35, C83.36, C83.37, C83.38, C83.39, C83.5, C83.50, C83.51, C83.52, C83.53, C83.54, C83.55, C83.56, C83.57, C83.58, C83.59, C83.7, C83.70, C83.71, C83.72, C83.73, C83.74, C83.75, C83.76, C83.77, C83.78, C83.79, C83.8, C83.80, C83.81, C83.82, C83.83, C83.84, C83.85, C83.86, C83.87, C83.88, C83.89, C83.9, C83.90, C83.91, C83.92, C83.93, C83.94, C83.95, C83.96, C83.97, C83.98, C83.99, C84, C84.0, C84.00, C84.01, C84.02, C84.03, C84.04, C84.05, C84.06, C84.07, C84.08, C84.09, C84.1, C84.10, C84.11, C84.12, C84.13, C84.14, C84.15, C84.16, C84.17, C84.18, C84.19, C84.4, C84.40, C84.41, C84.42, C84.43, C84.44, C84.45, C84.46, C84.47, C84.48, C84.49, C84.6, C84.60, C84.61, C84.62, C84.63, C84.64, C84.65, C84.66, C84.67, C84.68, C84.69, C84.7, C84.70, C84.71, C84.72, C84.73, C84.74, C84.75, C84.76, C84.77, C84.78, C84.79, C84.A, C84.A0, C84.A1, C84.A2, C84.A3, C84.A4, C84.A5, C84.A6, C84.A7, C84.A8, C84.A9, C84.Z, C84.Z0, C84.Z1, C84.Z2, C84.Z3, C84.Z4, C84.Z5, C84.Z6, C84.Z7, C84.Z8, C84.Z9, C84.9, C84.90, C84.91, C84.92, C84.93, C84.94, C84.95, C84.96, C84.97, C84.98, C84.99, C85, C85.1, C85.10, C85.11, C85.12, C85.13, C85.14, C85.15, C85.16, C85.17, C85.18, C85.19, C85.2, C85.20, C85.21, C85.22, C85.23, C85.24, C85.25, C85.26, C85.27, C85.28, C85.29, C85.8, C85.80, C85.81, C85.82, C85.83, C85.84, C85.85, C85.86, C85.87, C85.88, C85.89, C85.9, C85.90, C85.91, C85.92, C85.93, C85.94, C85.95, C85.96, C85.97, C85.98, C85.99, C86, C86.0, C86.1, C86.2, C86.3, C86.4, C86.5, C86.6, C88, C88.0, C88.2, C88.3, C88.4, C88.8, C88.9, C90, C90.0, C90.00, C90.01, C90.02, C90.1, C90.10, C90.11, C90.12, C90.2, C90.20, C90.21, C90.22, C90.3, C90.30, C90.31, C90.32, C91, C91.0, C91.00, C91.01, C91.02, C91.1, C91.10, C91.11, C91.12, C91.3, C91.30, C91.31, C91.32, C91.4, C91.40, C91.41, C91.42, C91.5, C91.50, C91.51, C91.52, C91.6, C91.60, C91.61, C91.62, C91.A, C91.A0, C91.A1, C91.A2, C91.Z, C91.Z0, C91.Z1, C91.Z2, C91.9, C91.90, C91.91, C91.92, C92 C92.0, C92.00, C92.01, C92.02, C92.1, C92.10, C92.11, C92.12, C92.2, C92.20, C92.21, C92.22, C92.3, C92.30, C92.31, C92.32, C92.4, C92.40, C92.41, C92.42, C92.5, C92.50, C92.51, C92.52, C92.6, C92.60, C92.61, C92.62, C92.A, C92.A0, C92.A1, C92.A2, C92.Z, C92.Z0, C92.Z1, C92.Z2, C92.9, C92.90, C92.91, C92.92, C93, C93.0, C93.00, C93.01, C93.02, C93.1, C93.10, C93.11, C93.12, C93.3, C93.30, C93.31, C93.32, C93.Z, C93.Z0, C93.Z1, C93.Z2, C93.9,

Variable	Definition	
		C93.90, C93.91, C93.92, C94, C94.0, C94.00, C94.01, C94.02, C94.2, C94.20, C94.21, C94.22, C94.3, C94.30, C94.31, C94.32, C94.4, C94.40, C94.41, C94.42, C94.6, C94.8, C94.80, C94.81, C94.82, C95, C95.0, C95.00, C95.01, C95.02, C95.1, C95.10, C95.11, C95.12, C95.9, C95.90, C95.91, C95.92, C96, C96.0, C96.2, C96.20, C96.21, C96.22, C96.29, C96.4, C96.5, C96.6, C96.A
Current smoker	Corporate Data Warehouse health factors file and existing algorithm	ICD-10 codes, at least one code in the past year<br>F17.200, F17.210, F17.290, Z72.0<br><br>Tobacco use CPT code, at least one code in the past year:<br>99406, 99407, S9075,<br>S9453, G0436, G0437<br><br>Clinic stop code, at least one code in the past year:<br>707, 708<br><br>OR<br><br>Most recent health factor within past year indicates current smoker. Table of smoking-related health factors adapted from table available at:<br>https://medicine.yale.edu/intmed/vacs/
Alcohol Use Disorder	Any single ICD code in past year	F10.10, F10.120, F10.121, F10.129, F10.14, F10.150, F10.151, F10.159, F10.180, F10.181, F10.182, F10.188, F10.19, F10.20, F10.220, F10.221, F10.229, F10.230, F10.231, F10.232, F10.239, F10.24, F10.250, F10.251, F10.259, F10.26, F10.27, F10.280, F10.281, F10.282, F10.288, F10.29, F10.920, F10.921, F10.929, F10.94, F10.950, F10.951, F10.959, F10.96, F10.97, F10.980, F10.981, F10.982, F10.988, F10.99, G31.2, G62.1, I42.6, K29.20, K29.21, K70.0, K70.10, K70.11, K70.2, K70.30, K70.31, K70.40, K70.41, K70.9, O35.4XX0, O35.4XX1, O35.4XX2, O35.4XX3, O35.4XX4, O35.4XX5, O35.4XX9, O99.310, O99.311, O99.312, O99.313, O99.314, O99.315, Z71.4, Z71.41
Drug Use Disorder	Any single ICD code in past year	F11.10, F11.120, F11.121, F11.122, F11.129, F11.13, F11.14, F11.150, F11.151, F11.159, F11.181, F11.182, F11.188, F11.19, F11.20, F11.21, F11.220, F11.221, F11.222, F11.229, F11.23, F11.24, F11.250, F11.251, F11.259, F11.281, F11.282, F11.288, F11.29, F11.90, F11.920, F11.921, F11.922, F11.929, F11.93, F11.94, F11.950, F11.951, F11.959, F11.981, F11.982, F11.988, F11.99, F12.10, F12.120, F12.121, F12.122, F12.129, F12.150, F12.151, F12.159, F12.180, F12.188, F12.19, F12.20, F12.21, F12.220, F12.221, F12.222, F12.229, F12.23, F12.250, F12.251, F12.259, F12.280, F12.288, F12.29, F12.90, F12.920, F12.921, F12.922, F12.929, F12.93, F12.950, F12.951, F12.959, F12.980, F12.988, F12.99, F13.10, F13.120, F13.121, F13.129, F13.14, F13.150, F13.151, F13.159, F13.180, F13.181, F13.182, F13.188, F13.19, F13.20, F13.21, F13.220, F13.221, F13.229, F13.230, F13.231, F13.232, F13.239, F13.24, F13.250, F13.251, F13.259, F13.26, F13.27, F13.280, F13.281, F13.282, F13.288, F13.29, F13.90, F13.920, F13.921, F13.929, F13.930, F13.931, F13.932, F13.939, F13.94, F13.950, F13.951, F13.959, F13.96, F13.97, F13.980, F13.981, F13.982, F13.988, F13.99, F14.10, F14.120, F14.121, F14.122, F14.129, F14.13, F14.14, F14.150, F14.151, F14.159, F14.180, F14.181, F14.182, F14.188, F14.19, F14.20, F14.21, F14.220, F14.221, F14.222, F14.229, F14.23, F14.24, F14.250, F14.251, F14.259, F14.280, F14.281, F14.282, F14.288, F14.29, F14.90, F14.920, F14.921, F14.922, F14.929, F14.93, F14.94, F14.950, F14.951, F14.959, F14.980, F14.981, F14.982, F14.988, F14.99, F15.10, F15.120, F15.121,

Variable	Definition	
		F15.122, F15.129, F15.13, F15.14, F15.150, F15.151, F15.182, F15.188, F15.19, F15.20, F15.21, F15.220, F15.221, F15.222, F15.229, F15.23, F15.24, F15.250, F15.251, F15.259, F15.280, F15.281, F15.282, F15.288, F15.29, F15.90, F15.920, F15.921, F15.922, F15.929, F15.93, F15.94, F15.950, F15.951, F15.959, F15.980, F15.981, F15.982, F15.988, F15.99, F16.10, F16.120, F16.121, F16.122, F16.129, F16.14, F16.150, F16.151, F16.159, F16.180, F16.183, F16.188, F16.19, F16.20, F16.21, F16.220, F16.221, F16.229, F16.24, F16.250, F16.251, F16.259, F16.280, F16.283, F16.288, F16.29, F16.90, F16.920, F16.921, F16.929, F16.94, F16.950, F16.951, F16.959, F16.980, F16.983, F16.988, F16.99, F18.10, F18.120, F18.121, F18.129, F18.14, F18.150, F18.151, F18.159, F18.17, F18.180, F18.188, F18.19, F18.20, F18.21, F18.220, F18.221, F18.229, F18.24, F18.250, F18.251, F18.259, F18.27, F18.280, F18.288, F18.29, F18.90, F18.920, F18.921, F18.929, F18.94, F18.950, F18.951, F18.959, F18.97, F18.980, F18.988, F18.99, F19.10, F19.120, F19.121, F19.122, F19.129, F19.130, F19.131, F19.132, F19.139, F19.14, F19.150, F19.151, F19.159, F19.16, F19.17, F19.180, F19.181, F19.182, F19.188, F19.19, F19.20, F19.21, F19.220, F19.221, F19.222, F19.229, F19.230, F19.231, F19.232, F19.239, F19.24, F19.250, F19.251, F19.259, F19.26, F19.27, F19.280, F19.281, F19.282, F19.288, F19.29, F19.90, F19.920, F19.921, F19.922, F19.929, F19.930, F19.931, F19.932, F19.939, F19.94, F19.950, F19.951, F19.959, F19.96, F19.97, F19.980, F19.981, F19.982, F19.988, F19.99
Marginally Housed	ICD codes or clinic stop codes related to marginal housing	ICD-10 codes, at least one code in past two years Z59.0 Homelessness Z59.1 Inadequate housing Z59.8 Other problems related to housing and economic circumstances Z59.9 Problem related to housing and economic circumstances, unspecified OR<br>Clinic Stop codes, at least one code in past two years: 507, 522, 528, 529, 530
Body Mass Index	Used more recent measurement before index date available in the Corporate Data Warehouse	
VA Priority score	VA Priority Groups	https://www.va.gov/health-care/eligibility/priority-groups/

Immunocompromised status defined based on receipt of three classes of drugs

(1) Receipt IV chemotherapy in the 90 days before vaccination completion
(2) Receipt of immunosuppressive drugs
(3) Receipt of antiretroviral drugs

Chemotherapy	Bendamustine, Busulfan, Carboplatin, Carmustine, Chlorambucil, Cisplatin, Cyclophosphamide, Dacarbazine, Estramustine, Ifosfamide, Lomustine, Mechlorethamine, Melphalan, Oxaliplatin, Pipobroman, Procarbazine, Streptozocin, Temozolomide, Thiotepa, Uracil Mustard, Azathioprine, Capecitabine, Cladribine, Clofarabine, Cytarabine, Floxuridine, Fludarabine, Fluorouracil, Gemcitabine, Hydroxyurea, Mercaptopurine, Methotrexate, Pentostatin, Pralatrexate, Thioguanine, Vidarabine, Daunorubicin, Doxorubicin, Epirubicin, Idarubicin, Pixantrone, Valrubicin, Vincristine, Vinblastine, Vinorelbine, Etoposide, Teniposide, Irinotecan, Mitoxantrone, Topotecan, Paclitaxel, Docetaxel
Immunosuppressive drugs	Abatacept, Adalimumab, Anakinra, Belimumab, Canakinumab, Certolizumab, Denosumab, Eculizumab, Etanercept, Golimumab, Infliximab, Natalizumab, Rituximab, Secukinumab,<br>Siltuximab, Tocilizumab, Sarilumab, Ustekinumab, Vedolizumab, Tofacitinib, Baricitinib, Upadacitinib, Apremilast, Mercaptopurine, Azathioprine, Cyclophosphamide, Cyclosporine, Hydroxychloroquine, Chloroquine, Leflunomide, Methotrexate, Mycophenolate mofetil, Mycophenolic acid, Sulfasalazine, Tacrolimus, Chloroquine, Hydroxychloroquine, Prednisone, Dexamethasone, Hydrocortisone, Methylprednisolone, Cortisone, Prednisolone, Triamcinolone, Betamethasone
Antiretroviral drugs	Abacavir (ABC), Didanosine (ddI), Emtricitabine (FTC), Lamivudine (3TC), Stavudine (d4T), Tenofovir (TDF), Zalcitabine (ddC), Zidovudine (ZDV, AZT), Delavirdine (DLV), Efavirenz (EFV), Etravirine (ETR), Nevirapine (NVP), Rilpivirine (RPV), Amprenavir (APV), Atazanavir (ATV), Atazanavir-cobicistat (ATV/COBI), Darunavir (DRV), Darunavir-cobicistat (DRV/COBI), Fosamprenavir (FPV), Indinavir (IDV), Lopinavir/ritonavir (LPV/r), Nelfinavir (NFV), Ritonavir (RTV), Saquinavir (SQV), Tipranavir (TPV), Enfuvirtide (T-20), Dolutegravir (DTG), Elvitegravir (EVG), Raltegravir (RAL), Maraviroc (MVC)

Supplementary Table 1: Expanded table of characteristics of the boosted cohort by vaccination series.

	Overall	mRNA-1273 x3	BNT162b2 x3
N	1,703,189	917,954	785,235
Male, n (%)	1,566,828 (92.0)	852,107 (92.8)	714,721 (91.0)
Female, n (%)	136,361 (8.0)	65,847 (7.2)	70,514 (9.0)
Age (median [IQR])	72 [63.0, 76.0]	72 [64.0, 77.0]	71 [61.0, 76.0]
18-34, n (%)	25,570 (1.5)	10,609 (1.2)	14,961 (1.9)
35-49, n (%)	115,597 (6.8)	51,286 (5.6)	64,311 (8.2)
50-64, n (%)	356,321 (20.9)	179,032 (19.5)	177,289 (22.6)
65-74, n (%)	625,953 (36.8)	341,048 (37.2)	284,905 (36.3)
75-84, n (%)	437,356 (25.7)	250,490 (27.3)	186,866 (23.8)
85 or older, n (%)	142,392 (8.4)	85,489 (9.3)	56,903 (7.2)
Race[a], n (%)			
American Indian or Alaska Native	11,220 (0.7)	5,995 (0.7)	5,225 (0.7)
Asian	24,503 (1.4)	11,757 (1.3)	12,746 (1.6)
Black or African American	330,079 (19.4)	148,949 (16.2)	181,130 (23.1)
More than one race	13,469 (0.8)	6,939 (0.8)	6,530 (0.8)
Native Hawaiian or Other Pacific Islander	15,155 (0.9)	7,812 (0.9)	7,343 (0.9)
Unknown Race	108,998 (6.4)	57,135 (6.2)	51,863 (6.6)
White	1,199,765 (70.4)	679,367 (74.0)	520,398 (66.3)
Hispanic or Latino ethnicity (regardless of race), n (%)	120,447 (7.1)	66,661 (7.3)	53,786 (6.8)
Currently Married, n (%)	1,018,655 (59.8)	560,871 (61.1)	457,784 (58.3)
Urban[b], n (%)	1,170,612 (68.7)	571,598 (62.3)	599,014 (76.3)
BMI (median [IQR])	29 [26.0, 33.3]	29 [26.0, 33.3]	29 [26.1, 33.4]
< 18.5, n (%)	11,710 (0.7)	6,275 (0.7)	5,435 (0.7)
18.5 - 24.9, n (%)	280,883 (16.5)	151,927 (16.6)	128,956 (16.4)
25 - 29.9, n (%)	589,295 (34.6)	316,639 (34.5)	272,656 (34.7)
>= 30, n (%)	721,252 (42.3)	385,192 (42.0)	336,060 (42.8)
Unknown, n (%)	100,049 (5.9)	57,921 (6.3)	42,128 (5.4)
Comorbidities associated with severe COVID-19 illness, n (%)			
Hypertension	1,043,181 (61.2)	578,501 (63.0)	464,680 (59.2)
Diabetes	554,540 (32.6)	308,289 (33.6)	246,251 (31.4)
CKD[c]	355,021 (20.8)	199,994 (21.8)	155,027 (19.7)
IHD	291,422 (17.1)	166,077 (18.1)	125,345 (16.0)
COPD Bronchiectasis	196,384 (11.5)	114,691 (12.5)	81,693 (10.4)
CHF	96,188 (5.6)	53,640 (5.8)	42,548 (5.4)
Immunocompromised[d]	120,111 (7.1)	64,316 (7.0)	55,795 (7.1)
Cancer solid organ[e]	59,172 (3.5)	31,527 (3.4)	27,645 (3.5)
Severe CKD[f]	43,843 (2.6)	26,242 (2.9)	17,601 (2.2)
Stroke, TIA	45,239 (2.7)	24,295 (2.6)	20,944 (2.7)
Dementia	32,089 (1.9)	18,086 (2.0)	14,003 (1.8)
Cirrhosis	26,480 (1.6)	13,133 (1.4)	13,347 (1.7)
Cancer lymphoma leukemia[e]	21,103 (1.2)	11,804 (1.3)	9,299 (1.2)

	Overall	mRNA-1273 x3	BNT162b2 x3
Dialysis	12,780 (0.8)	6,924 (0.8)	5,856 (0.7)
Cancer, other[e]	10,752 (0.6)	5,425 (0.6)	5,327 (0.7)
SCI clinic	9,840 (0.6)	4,891 (0.5)	4,949 (0.6)
Home based primary care	26,842 (1.6)	16,429 (1.8)	10,413 (1.3)
History of COVID-19 infection[g]	83,872 (4.9)	42,274 (4.6)	41,598 (5.3)
Primary quarter, n (%)			
12/2020 – 02/2021	1,117,531 (65.6)	624,545 (68.0)	492,986 (62.8)
03/2021 – 05/2021	560,429 (32.9)	282,717 (30.8)	277,712 (35.4)
06/2021 – 08/2021	22,708 (1.3)	9,782 (1.1)	12,926 (1.6)
09/2021 – 11/2021	2,513 (0.1)	907 (0.1)	1,606 (0.2)
12/2021 – 02/2022	8 (0.0)	3 (0.0)	5 (0.0)
Current smoker	352,343 (20.7)	190,127 (20.7)	162,216 (20.7)
Alcohol abuse[h]	118,757 (7.0)	61,581 (6.7)	57,176 (7.3)
Substance use[i]	74,262 (4.4)	37,537 (4.1)	36,725 (4.7)
Homelessness[j]	60,951 (3.6)	30,528 (3.3)	30,423 (3.9)
Follow up (median [IQR])	188 [163.0, 213.0]	180 [159.0, 202.0]	199 [168.0, 224.0]
Time between (median [IQR])	270 [246.0, 295.0]	280 [258.0, 301.0]	258 [237.0, 282.0]
High-risk populations	1,592,704 (93.5)	867,665 (94.5)	725,039 (92.3)
Age $\geq$65 and no high-risk co-morbid conditions, n (%)	215,177 (12.6)	118,313 (12.9)	96,864 (12.3)
High risk not immunocompromised[d], n (%)	1,188,470 (69.8)	647,603 (70.5)	540,867 (68.9)
Immunocompromised, n (%)	189,057 (11.1)	101,749 (11.1)	87,308 (11.1)
Age <65 and no co-morbid conditions, n (%)	110,485 (6.5)	50,289 (5.5)	60,196 (7.7)
CAN[k] score, n (%)			
0 - 49.9	583,130 (34.2)	292,460 (31.9)	290,670 (37.0)
50 - 74.9	533,079 (31.3)	293,259 (31.9)	239,820 (30.5)
75 - 100	586,980 (34.5)	332,235 (36.2)	254,745 (32.4)

[a]Race/ethnicity was assessed using self-identified data found in Veteran Health Records.
[b]Urban/rural was assessed using defined based on the Rural Urban Commuting Area (RUCA) categories developed by the Department of Agriculture and Health and Human Services' Health Resource and Services Administration.
[c]CKD defined as having a glomerular filtration rate between 30 and 60.
[d]Immunocompromised definition based on medications and history of cancer (see supplement for list of meds).
[e]Cancer definition based on diagnosis codes. 2 outpatient or 1 inpatient diagnosis code in the VHA (see supplement).
[f]Severe CKD defined as having a glomerular filtration rate <30.
[g]91 or more days prior to boosted shot
[h]Alcohol use disorder defined as 1 outpatient or 1 inpatient code within 2 years of index.
[i]Including cannabis, opioids, inhalants.
[j]Housing problems defined as homelessness, inadequate housing, other problems related to housing and economic circumstances.
[k]The Care Assessment Need (CAN) Score is a predictive analytic tool that estimates the relative probability of hospitalization and death within 90 days or one-year from the calculation date. The oOffice of Clinical Systems Development and Evaluation (CSDE-10E2A) produces the weekly CAN Score Report to help identify the highest risk patients in a primary care panel or cohort. We used the one-year score.

Abbreviations: BMI: body mass index; CHF: chronic heart failure; CKD: chronic kidney disease; COPD: chronic obstructive pulmonary disease; TIA: transient ischemic attack

Supplementary Table 2: Characteristics of cohort by outcome.

	Breakthrough COVID-19		Hospitalization for COVID-19 pneumonia/death	
	No	Yes	No	Yes
N	1,680,341	22,848	1,701,540	1,649
Male, n (%)	1,546,397 (92.0)	20,431 (89.4)	1,565,240 (92.0)	1,588 (96.3)
Female, n (%)	133,944 (8.0)	2,417 (10.6)	136,300 (8.0)	61 (3.7)
Age, median (q1, q3)	72 [63.0, 76.0]	69 [59.0, 75.0]	72 [62.0, 76.0]	74 [70.0, 81.0]
Age, n (%)				
18-34	25,148 (1.5)	422 (1.8)	25,569 (1.5)	1 (0.1)
35-49	113,399 (6.7)	2,198 (9.6)	115,587 (6.8)	10 (0.6)
50-64	350,332 (20.8)	5,989 (26.2)	356,135 (20.9)	186 (11.3)
65-74	617,857 (36.8)	8,096 (35.4)	625,324 (36.8)	629 (38.1)
75-84	432,597 (25.7)	4,759 (20.8)	436,831 (25.7)	525 (31.8)
85 or older	141,008 (8.4)	1,384 (6.1)	142,094 (8.4)	298 (18.1)
Race[a], n (%)				
American Indian or Alaska Native	11,040 (0.7)	180 (0.8)	11,207 (0.7)	13 (0.8)
Asian	24,136 (1.4)	367 (1.6)	24,498 (1.4)	5 (0.3)
Black or African American	324,688 (19.3)	5,391 (23.6)	329,779 (19.4)	300 (18.2)
Missing	107,635 (6.4)	1,363 (6.0)	108,909 (6.4)	89 (5.4)
More than one race	13,269 (0.8)	200 (0.9)	13,454 (0.8)	15 (0.9)
Native Hawaiian or other Pacific Islander	14,920 (0.9)	235.0 (1.0)	15,142 (0.9)	13 (0.8)
White	1,184,653 (70.5)	15,112 (66.1)	1,198,551 (70.4)	1,214 (73.6)
Hispanic or Latino Ethnicity (regardless of race), n (%)	117,948 (7.0)	2,499 (10.9)	120,335 (7.1)	112 (6.8)
Married, n (%)	1,006,077 (59.9)	12,578 (55.1)	1,017,714 (59.8)	941 (57.1)
Urban[b], n (%)	1,152,573 (68.6)	18,039 (79.0)	1,169,441 (68.7)	1,171 (71.0)
BMI, median (q1, q3)	29 [26.0, 33.3]	30 [26.3, 33.9]	29 [26.0, 33.3]	28.53 [25.0, 33.0]
BMI, n (%)				
< 18.5	11,572 (0.7)	138 (0.6)	11,682 (0.7)	28 (1.7)
18.5 - 24.9	277,293 (16.5)	3,590 (15.7)	280,509 (16.5)	374 (22.7)
25 - 29.9	581,511 (34.6)	7,784 (34.1)	588,733 (34.6)	562 (34.1)
>= 30	710,583 (42.3)	10,669 (46.7)	720,612 (42.4)	640 (38.8)
Unknown	99,382 (5.9)	667 (2.9)	100,004 (5.9)	45 (2.7)
Hypertension, n (%)	1,027,732 (61.2)	15,449 (67.6)	1,041,804 (61.2)	1,377 (83.5)
Diabetes, n (%)	545,850 (32.5)	8,690 (38.0)	553,667 (32.5)	873 (52.9)
CKD[c], n (%)	350,399 (20.9)	4,622 (20.2)	354,460 (20.8)	561 (34.0)
Ischemic Heart Disease, n (%)	286,294 (17.0)	5,128 (22.4)	290,770 (17.1)	652 (39.5)
COPD Bronchiectasis, n (%)	192,606 (11.5)	3,778 (16.5)	195,790 (11.5)	594 (36.0)
CHF, n (%)	93,762 (5.6)	2,426 (10.6)	95,711 (5.6)	477 (28.9)
Immunocompromised[d], n (%)	116,485 (6.9)	3,626 (15.9)	119,609 (7.0)	502 (30.4)
Cancer[e] - solid organ, n (%)	57,930 (3.4)	1,242 (5.4)	58,991 (3.5)	181 (11.0)
Severe CKD[f], n (%)	42,800 (2.5)	1,043 (4.6)	43,584 (2.6)	259 (15.7)
Stroke TIA, n (%)	44,271 (2.6)	968 (4.2)	45,118 (2.7)	121 (7.3)
Dementia, n (%)	31,399 (1.9)	690 (3.0)	31,956 (1.9)	133 (8.1)
Cirrhosis, n (%)	25,796 (1.5)	684 (3.0)	26,387 (1.6)	93 (5.6)
Cancer[e] - lymphoma leukemia, n (%)	20,413 (1.2)	690 (3.0)	20,921 (1.2)	182 (11.0)
Dialysis, n (%)	12,193 (0.7)	587 (2.6)	12,608 (0.7)	172 (10.4)
Cancer[e] - other, n (%)	10,448 (0.6)	304 (1.3)	10,699 (0.6)	53 (3.2)
Spinal cord injury, n (%)	9,593 (0.6)	247 (1.1)	9,812 (0.6)	28 (1.7)
Home based primary care, n (%)	26,155 (1.6)	687 (3.0)	26,676 (1.6)	166 (10.1)
Prior history of COVID-19 infection[g], n (%)	83,314 (5.0)	558 (2.4)	83,792 (4.9)	80 (4.9)
Date of first dose of vaccination series, by 3-month period, n (%)				
12/2020 - 02/2021	1,101,802 (65.6)	15,729 (68.8)	1,116,177 (65.6)	1,354 (82.1)

	Breakthrough COVID-19		Hospitalization for COVID-19 pneumonia/death	
03/2021 - 05/2021	553,425 (32.9)	7,004 (30.7)	560,136 (32.9)	293 (17.8)
06/2021 - 08/2021	22,596 (1.3)	112 (0.5)	22,706 (1.3)	2 (0.1)
09/2021 - 11/2021	2,510 (0.1)	3 (0.0)	2,513 (0.1)	0 (0.0)
12/2021 - 02/2022	8 (0.0)	0 (0.0)	8 (0.0)	0 (0.0)
Current smoker, n (%)	348,118 (20.7)	4,225 (18.5)	352,053 (20.7)	290 (17.6)
Alcohol use disorder[h], n (%)	116,657 (6.9)	2,100 (9.2)	118,632 (7.0)	125 (7.6)
Substance use disorder[i], n (%)	72,745 (4.3)	1,517 (6.6)	74,177 (4.4)	85 (5.2)
Housing problems[j], n (%)	59,582 (3.5)	1,369 (6.0)	60,869 (3.6)	82 (5.0)
Median follow-up time (IQR), days	188 [163.0, 213.0]	194 [166.0, 217.0]	188 [163.0, 213.0]	93 [54.0, 134.0]
Median time elapsed between initial dose and booster (IQR), days	270 [246.0, 295.0]	265 [241.0, 289.0]	270 [246.0, 295.0]	260 [232.0, 289.0]
High-risk populations, n (%)	1,571,518 (93.5)	21,186 (92.7)	1,591,067 (93.5)	1,637 (99.3)
Age > 65 and no high-risk co-morbid conditions, n (%)	213,556 (12.7)	1,621 (7.1)	215,129 (12.6)	48 (2.9)
High-risk co-morbid conditions (not immunocompromised), n (%)	1,173,966 (69.9)	14,504 (63.5)	1,187,625 (69.8)	845 (51.2)
Immunocompromised[d], n (%)	183,996 (10.9)	5,061 (22.2)	188,313 (11.1)	744 (45.1)
Age < 65 and no co-morbid conditions, n (%)	108,823 (6.5)	1,662 (7.3)	110,473 (6.5)	12 (0.7)
CAN[k] score, n (%)				
0 - 49.9	574,784 (34.2)	8,346 (36.5)	583,092 (34.3)	38 (2.3)
50 - 74.9	527,287 (31.4)	5,792 (25.4)	532,928 (31.3)	151 (9.2)
75 - 100	578,270 (34.4)	8,710 (38.1)	585,520 (34.4)	1,460 (88.5)

[a]Race/ethnicity was assessed using self-identified data found in Veteran Health Records.
[b]Urban/rural was assessed using defined based on the Rural Urban Commuting Area (RUCA) categories developed by the Department of Agriculture and Health and Human Services' Health Resource and Services Administration.
[c]CKD defined as having a glomerular filtration rate between 30 and 60.
[d]Immunocompromised definition based on medications and history of cancer (see supplement for list of meds).
[e]Cancer definition based on diagnosis codes. 2 outpatient or 1 inpatient diagnosis code in the VHA (see supplement).
[f]Severe CKD defined as having a glomerular filtration rate <30.
[g]91 or more days prior to boosted shot
[h]Alcohol use disorder defined as 1 outpatient or 1 inpatient code within 2 years of index.
[i]Including cannabis, opioids, inhalants.
[j]Housing problems defined as homelessness, inadequate housing, other problems related to housing and economic circumstances.
[k]The Care Assessment Need (CAN) Score is a predictive analytic tool that estimates the relative probability of hospitalization and death within 90 days or one-year from the calculation date. The oOffice of Clinical Systems Development and Evaluation (CSDE-10E2A) produces the weekly CAN Score Report to help identify the highest risk patients in a primary care panel or cohort. We used the one-year score.

Abbreviations: BMI: body mass index; CHF: chronic heart failure; CKD: chronic kidney disease; COPD: chronic obstructive pulmonary disease; TIA: transient ischemic attack

Supplementary Table 3: Interaction analyses of booster type and hospitalization with COVID-19 pneumonia or death by Delta vs Omicron variant eras.

Breakthrough COVID							
	Delta			Omicron			
	Patients	Outcomes	Cumulative Incidence Ratio (95% CI)	Patients	Outcomes	Cumulative Incidence Ratio (95% CI)	Interaction p
**mRNA-1273 x3**	549,623	320	ref	228,307	1,957	ref	ref
**BNT162b2 x3**	550,185	450	1.4 (1.1, 1.7)	160,798	1,590	1.2 (0.9, 1.5)	0.30
**COVID-19 pneumonia**							
	Delta			Omicron			
	Patients	Outcomes	Cumulative Incidence Ratio (95% CI)	Patients	Outcomes	Cumulative Incidence Ratio (95% CI)	Interaction p
**mRNA-1273 x3**	549,623	68	ref	228,307	110	ref	ref
**BNT162b2 x3**	550,185	98	1.6 (1.1, 2.3)	160,798	74	1.3 (0.9, 1.9)	0.45

Supplementary Tables 4-9: Propensity weighted samples of veterans, overall and by sub-group, who received three doses of mRNA-1273 or BNT-162b2 COVID-19 vaccines.

Supplementary Table 4: Propensity weighted samples of all veterans who received three doses of mRNA-1273 or BNT-162b2 COVID-19 vaccines.

	mRNA-1273 x3 Unweighted	BNT162b2 x3 Unweighted	mRNA-1273 x3 Weighted	BNT162b2 x3 Weighted
N	917,954	785,235	853,625	727,698
Male, n (%)	852,107 (92.8)	714,721.0 (91.0)	787,180.6 (92.2)	668,979.5 (91.9)
Female, n (%)	65,847 (7.2)	70,514.0 (9.0)	66,444.3 (7.8)	58,718.3 (8.1)
Age, n (%)	70 (12.1)	67.8 (13.1)	69.4 (12.6)	69.1 (12.8)
Race[a], n (%)				
White	679,367 (74.0)	520,398.0 (66.3)	617,486.5 (72.3)	515,102.0 (70.8)
Black or African American	148,949 (16.2)	181,130.0 (23.1)	148,378.9 (17.4)	136,443.0 (18.7)
Unknown	89,638 (9.8)	83,707.0 (10.7)	87,759.5 (10.3)	76,152.8 (10.5)
Hispanic or Latino Ethnicity (regardless of race), n (%)	66,661 (7.3)	53,786.0 (6.8)	59,861.0 (7.0)	50,798.8 (7.0)
Currently Married, n (%)	560,871 (61.1)	457,784.0 (58.3)	527,827.0 (61.8)	450,234.2 (61.9)
Highly Rural or unknown, n (%)	45,819 (5.0)	18,162.0 (2.3)	35,769.5 (4.2)	27,283.6 (3.7)
Rural, n (%)	300,537 (32.7)	168,059.0 (21.4)	258,398.2 (30.3)	197,754.6 (27.2)
Urban[b], n (%)	571,598 (62.3)	599,014.0 (76.3)	559,457.2 (65.5)	502,659.5 (69.1)
VHA Priority, n (%)				
1-4	578,035 (63.0)	511,266.0 (65.1)	543,421.1 (63.7)	465,660.0 (64.0)
5-6	177,325 (19.3)	141,888.0 (18.1)	158,219.3 (18.5)	131,939.4 (18.1)
7-8	162,594 (17.7)	132,081.0 (16.8)	151,984.5 (17.8)	130,098.4 (17.9)
Reinfection[c], n (%)	42,274 (4.6)	41,598.0 (5.3)	38,734.4 (4.5)	34,005.8 (4.7)
BMI, n (%)				
<18.5	6,275 (0.7)	5,435.0 (0.7)	5,640.7 (0.7)	4,752.5 (0.7)
18.5-24.9	151,927 (16.6)	128,956.0 (16.4)	139,867.1 (16.4)	118,961.9 (16.3)
25-29.9	316,639 (34.5)	272,656.0 (34.7)	296,040.5 (34.7)	253,674.2 (34.9)
>=30	385,192 (42.0)	336,060.0 (42.8)	358,971.6 (42.1)	305,944.9 (42.0)
BMI unknown	57,921 (6.3)	42,128.0 (5.4)	53,105.1 (6.2)	44,364.4 (6.1)
Comorbidities associated with severe COVID-19 illness, n (%)				
Hypertension	578,501 (63.0)	464,680.0 (59.2)	521,298.8 (61.1)	436,634.4 (60.0)
CHF	53,640 (5.8)	42,548.0 (5.4)	45,946.3 (5.4)	37,554.6 (5.2)
IHD	166,077 (18.1)	125,345.0 (16.0)	147,163.5 (17.2)	121,389.6 (16.7)
Diabetes	308,289 (33.6)	246,251.0 (31.4)	275,853.8 (32.3)	229,979.1 (31.6)
Stroke TIA	24,295 (2.6)	20,944.0 (2.7)	21,594.0 (2.5)	18,101.8 (2.5)
COPD Bronchiectasis	114,691 (12.5)	81,693.0 (10.4)	97,445.5 (11.4)	77,983.5 (10.7)
Cirrhosis	13,133 (1.4)	13,347.0 (1.7)	12,025.8 (1.4)	10,385.5 (1.4)
Dementia	18,086 (2.0)	14,003.0 (1.8)	16,164.5 (1.9)	13,549.3 (1.9)
Immunocompromised[d]	64,316 (7.0)	55,795.0 (7.1)	56,515.0 (6.6)	47,288.8 (6.5)

	mRNA-1273 x3 Unweighted	BNT162b2 x3 Unweighted	mRNA-1273 x3 Weighted	BNT162b2 x3 Weighted
Spinal cord injury	4,891 (0.5)	4,949.0 (0.6)	4,535.2 (0.5)	3,926.7 (0.5)
No CKD[e]	691,718 (75.4)	612,607.0 (78.0)	651,286.6 (76.3)	558,430.0 (76.7)
CKD[e]	199,994 (21.8)	155,027.0 (19.7)	180,525.8 (21.1)	151,255.1 (20.8)
Severe CKD[f]	26,242 (2.9)	17,601.0 (2.2)	21,812.5 (2.6)	18,012.7 (2.5)
Dialysis	6,924 (0.8)	5,856.0 (0.7)	6,092.3 (0.7)	5,079.4 (0.7)
Cancer[g] solid organ	31,527 (3.4)	27,645.0 (3.5)	28,044.1 (3.3)	23,532.8 (3.2)
Cancer[g] lymphoma leukemia	11,804 (1.3)	9,299.0 (1.2)	10,331.5 (1.2)	8,472.9 (1.2)
Cancer[g] other	5,425 (0.6)	5,327.0 (0.7)	4,872.2 (0.6)	4,296.4 (0.6)
Home based primary care, n (%)	16,429 (1.8)	10,413.0 (1.3)	13,093.6 (1.5)	9,813.5 (1.3)
Current smoker, n (%)	190,127 (20.7)	162,216.0 (20.7)	170,825.6 (20.0)	142,689.4 (19.6)
Alcohol use disorder[h], n (%)	61,581 (6.7)	57,176.0 (7.3)	55,641.5 (6.5)	47,248.4 (6.5)
Substance use disorder[i], n (%)	37,537 (4.1)	36,725.0 (4.7)	33,150.3 (3.9)	28,410.6 (3.9)
Housing problems[j], n (%)	30,528 (3.3)	30,423.0 (3.9)	26,892.4 (3.2)	23,191.6 (3.2)
CAN[k], n (%)				
0-49.9	292,460 (31.9)	290,670.0 (37.0)	285,918.9 (33.5)	250,329.3 (34.4)
50-74.9	293,259 (31.9)	239,820.0 (30.5)	269,241.6 (31.5)	227,761.9 (31.3)
75-100	332,235 (36.2)	254,745.0 (32.4)	298,464.4 (35.0)	249,606.6 (34.3)
Primary quarter, n (%)				
12/2020-02/2021	624,545.0 (68.0)	492,986.0 (62.8)	572,480.9 (67.1)	482,011.7 (66.2)
03/2021-05/2021	282,717.0 (30.8)	277,712.0 (35.4)	268,681.3 (31.5)	233,697.8 (32.1)
06/2021-08/2021	9,782.0 (1.1)	12,926.0 (1.6)	11,345.6 (1.3)	10,812.6 (1.5)
09/2021-11/2021	907.0 (0.1)	1,606.0 (0.2)	1,113.1 (0.1)	1,172.6 (0.2)
12/2021-02/2022	3.0 (0.0)	5.0 (0.0)	3.9 (0.0)	3.0 (0.0)

[a]Race/ethnicity was assessed using self-identified data found in Veteran Health Records.
[b]Urban/rural was assessed using defined based on the Rural Urban Commuting Area (RUCA) categories developed by the Department of Agriculture and Health and Human Services' Health Resource and Services Administration.
[c]91 or more days prior to boosted shot
[d]Immunocompromised definition based on medications and history of cancer (see supplement for list of meds).
[e]CKD defined as having a glomerular filtration rate between 30 and 60.
[f]Severe CKD defined as having a glomerular filtration rate <30.
[g]Cancer definition based on diagnosis codes. 2 outpatient or 1 inpatient diagnosis code in the VHA (see supplement).
[h]Alcohol use disorder defined as 1 outpatient or 1 inpatient code within 2 years of index.
[i]Including cannabis, opioids, inhalants.
[j]Housing problems defined as homelessness, inadequate housing, other problems related to housing and economic circumstances.
[k]The Care Assessment Need (CAN) Score is a predictive analytic tool that estimates the relative probability of hospitalization and death within 90 days or one-year from the calculation date. The Office of Clinical Systems Development and Evaluation (CSDE-10E2A) produces the weekly CAN Score Report to help identify the highest risk patients in a primary care panel or cohort. We used the one-year score.

Abbreviations: BMI: body mass index; CHF: chronic heart failure; CKD: chronic kidney disease; COPD: chronic obstructive pulmonary disease; TIA: transient ischemic attack

Unweighted samples: Standardized differences were less than 0.14 for all variables.
Weighted samples: Standardized differences were less than 0.04 for all variables.

Supplementary Table 5: Propensity weighted samples of average-risk veterans (age ≤65 and no high-risk co-morbid conditions) who have received three doses of mRNA-1273 or BNT-162b2 COVID-19 vaccines.

	mRNA-1273 x3 Unweighted	BNT162b2 x3 Unweighted	mRNA-1273 x3 Weighted	BNT162b2 x3 Weighted
N	50,289	60,196	46,637	55,509
Male, n (%)	38,577.0 (76.7)	45,084.0 (74.9)	35,057.9 (75.2)	41,569.30 (74.9)
Female, n (%)	11,712.0 (23.3)	15,112.0 (25.1)	11,579.2 (24.8)	13,939.9 (25.1)
Age, n (%)	51.5 (10.3)	49.7 (10.6)	50.6 (10.5)	50.10 (10.6)
Race[a], n (%)				
White	31,548.0 (62.7)	33,881.0 (56.3)	28,168.9 (60.4)	32,575.30 (58.7)
Black or African American	10,987.0 (21.8)	15,698.0 (26.1)	10,537.2 (22.6)	13,244.90 (23.9)
Unknown	7,754.0 (15.4)	10,617.0 (17.6)	7,931.0 (17.0)	9,689 (17.5)
Hispanic or Latino Ethnicity (regardless of race), n (%)	6,291.0 (12.5)	6,520.0 (10.8)	5,438.9 (11.7)	6,526.9 (11.8)
Currently Married, n (%)	26,598.0 (52.9)	30,342.0 (50.4)	24,505.8 (52.5)	28,830.8 (51.9)
Highly Rural or unknown, n (%)	1,400.0 (2.8)	729.0 (1.2)	1,006.6 (2.2)	1,022.6 (1.8)
Rural, n (%)	11,631.0 (23.1)	9,058.0 (15.0)	9,487.1 (20.3)	10,059.7 (18.1)
Urban[b], n (%)	37,258.0 (74.1)	50,409.0 (83.7)	36,143.3 (77.5)	44,427 (80.0)
VHA Priority, n (%)				
1-4	39,076.0 (77.7)	47,705.0 (79.2)	36,683.5 (78.7)	43,816.6 (78.9)
5-6	5,230.0 (10.4)	5,495.0 (9.1)	4,462.0 (9.6)	5,153.9 (9.3)
7-8	5,983.0 (11.9)	6,996.0 (11.6)	5,491.6 (11.8)	6,538.7 (11.8)
Reinfection[c], n (%)	2,553.0 (5.1)	3,258.0 (5.4)	2,296.6 (4.9)	2,833.3 (5.1)
BMI, n (%)				
<18.5	230.0 (0.5)	327.0 (0.5)	232.1 (0.5)	290.3 (0.5)
18.5-24.9	11,547.0 (23.0)	14,214.0 (23.6)	10,915.8 (23.4)	12,926.3 (23.3)
25-29.9	31,929.0 (63.5)	38,036.0 (63.2)	29,331.6 (62.9)	34,939.7 (62.9)
>=30	6,583.0 (13.1)	7,619.0 (12.7)	6,157.6 (13.2)	7,353.0 (13.2)
BMI unknown	12,041.0 (23.9)	12,413.0 (20.6)	10,222.2 (21.9)	11,823.1 (21.3)
Comorbidities associated with severe COVID-19 illness, n (%)				
Hypertension	323.0 (0.6)	337.0 (0.6)	258.7 (0.6)	288.9 (0.5)
Stroke TIA	417.0 (0.8)	469.0 (0.8)	363.0 (0.8)	426.2 (0.8)
Spinal cord injury	14.0 (0.0)	19.0 (0.0)	14.6 (0.0)	16.6 (0.0)
Cancer[d] other	43.0 (0.1)	40.0 (0.1)	37.0 (0.1)	41.8 (0.1)
Home based primary care	189.0 (0.4)	126.0 (0.2)	130.1 (0.3)	134.1 (0.2)
Alcohol use disorder[e], n (%)	3,320.0 (6.6)	3,930.0 (6.5)	2,882.5 (6.2)	3,465.1 (6.2)
Substance use disorder[f], n (%)	2,053.0 (4.1)	2,285.0 (3.8)	1,702.9 (3.7)	1,990.0 (3.6)
Housing problems[g], n (%)	1,563.0 (3.1)	1,874.0 (3.1)	1,301.5 (2.8)	1,570.0 (2.8)
CAN[h], n (%)				
0-49.9	44,414.0 (88.3)	53,972.0 (89.7)	41,693.9 (89.4)	49,748.0 (89.6)
50-74.9	5,376.0 (10.7)	5,723.0 (9.5)	4,543.2 (9.7)	5,292.7 (9.5)
75-100	499.0 (1)	501.0 (0.8)	400.0 (0.9)	468.4 (0.8)
Primary quarter, n (%)				
12/2020-02/2021	18,045.0 (35.9)	18,023.0 (29.9)	17,014.4 (36.5)	19,230.3 (34.6)
03/2021-05/2021	31,198.0 (62)	40,161.0 (66.7)	28,241.2 (60.6)	34,487.4 (62.1)
06/2021-08/2021	978.0 (1.9)	1,824.0 (3.0)	1,282.3 (2.7)	1,644.7 (3.0)
09/2021-11/2021	68.0 (0.1)	188.0 (0.3)	99.1 (0.2)	146.8 (0.3)

[a]Race/ethnicity was assessed using self-identified data found in Veteran Health Records.
[b]Urban/rural was assessed using defined based on the Rural Urban Commuting Area (RUCA) categories developed by the Department of Agriculture and Health and Human Services' Health Resource and Services Administration.
[c]91 or more days prior to booster shot
[d]Cancer definition based on diagnosis codes. 2 outpatient or 1 inpatient diagnosis code in the VHA (see supplement).
[e]Alcohol use disorder defined as 1 outpatient or 1 inpatient code within 2 years of index.
[f]Including cannabis, opioids, inhalants.
[g]Housing problems defined as homelessness, inadequate housing, other problems related to housing and economic circumstances.

[h]The Care Assessment Need (CAN) Score is a predictive analytic tool that estimates the relative probability of hospitalization and death within 90 days or one-year from the calculation date. The Office of Clinical Systems Development and Evaluation (CSDE-10E2A) produces the weekly CAN Score Report to help identify the highest risk patients in a primary care panel or cohort. We used the one-year score.

Abbreviations: BMI: body mass index; CHF: chronic heart failure; CKD: chronic kidney disease; COPD: chronic obstructive pulmonary disease; TIA: transient ischemic attack

Unweighted samples: Standardized differences were less than 0.14 for all variables.
Weighted samples: Standardized differences were less than 0.03 for all variables

Supplementary Table 6: Propensity weighted samples of high-risk veterans (age ≥65, high-risk co-morbid conditions, immunocompromising conditions) who have received three doses of mRNA-1273 or BNT-162b2 COVID-19 vaccines.

	mRNA-1273 x3 Unweighted	BNT162b2 x3 Unweighted	mRNA-1273 x3 Weighted	BNT162b2 x3 Weighted
N	867,665	725,039	806,855	671,546
Male, n (%)	813,530.0 (93.8)	669,637.0 (92.4)	753,128.1 (93.3)	625,432.3 (93.1)
Female, n (%)	54,135.0 (6.2)	55,402.0 (7.6)	53,727.0 (6.7)	46,114.2 (6.9)
Age, n (%)	71.1 (11.4)	69.3 (12.1)	70.7 (11.6)	70.4 (11.9)
Race[a], n (%)				
White	647,819.0 (74.7)	486,517.0 (67.1)	590,092.3 (73.1)	480,939.7 (71.6)
Black or African American	137,962.0 (15.9)	165,432.0 (22.8)	137,342.6 (17.0)	123,563.9 (18.4)
Unknown	81,884.0 (9.4)	73,090.0 (10.1)	79,420.2 (9.8)	67,042.9 (10.0)
Hispanic or Latino Ethnicity (regardless of race), n (%)	60,370.0 (7.0)	47,266.0 (6.5)	54,118.4 (6.7)	44,553.1 (6.6)
Currently Married, n (%)	534,273.0 (61.6)	427,442.0 (59.0)	503,767.8 (62.4)	420,146.7 (62.6)
Highly Rural or unknown, n (%)	44,419.0 (5.1)	17,433.0 (2.4)	34,944.8 (4.3)	26,127.2 (3.9)
Rural, n (%)	288,906.0 (33.3)	159,001.0 (21.9)	249,644.2 (30.9)	186,977.5 (27.8)
Urban[b], n (%)	534,340.0 (61.6)	548,605.0 (75.7)	522,266.1 (64.7)	458,441.8 (68.3)
VHA Priority, n (%)				
1-4	538,959.0 (62.1)	463,561.0 (63.9)	505,590.8 (62.7)	422,674.0 (62.9)
5-6	172,095.0 (19.8)	136,393.0 (18.8)	154,367.8 (19.1)	125,948.6 (18.8)
7-8	156,611.0 (18.0)	125,085.0 (17.3)	146,896.5 (18.2)	122,923.9 (18.3)
Reinfection[c], n (%)	39,721.0 (4.6)	38,340.0 (5.3)	36,433.0 (4.5)	31,217.9 (4.6)
BMI, n (%)				
<18.5	6,045.0 (0.7)	5,108.0 (0.7)	5,421.5 (0.7)	4,451.0 (0.7)
18.5-24.9	140,380.0 (16.2)	114,742.0 (15.8)	128,501.4 (15.9)	106,330.2 (15.8)
25-29.9	284,710.0 (32.8)	234,620.0 (32.4)	264,534.5 (32.8)	220,457.8 (32.8)
>=30	385,192.0 (44.4)	336,060.0 (46.4)	362,029.0 (44.9)	302,721.3 (45.1)
BMI unknown	51,338.0 (5.9)	34,509.0 (4.8)	46,368.8 (5.7)	37,586.2 (5.6)
Comorbidities associated with severe COVID-19 illness, n (%)				
Hypertension	566,460.0 (65.3)	452,267.0 (62.4)	513,840.3 (63.7)	421,347.7 (62.7)
CHF	53,640.0 (6.2)	42,548.0 (5.9)	46,346.0 (5.7)	37,121.9 (5.5)
IHD	166,077.0 (19.1)	125,345.0 (17.3)	148,391.3 (18.4)	119,951.7 (17.9)
Diabetes	308,289.0 (35.5)	246,251.0 (34.0)	278,227.2 (34.5)	227,408.9 (33.9)
Stroke TIA	23,972.0 (2.8)	20,607.0 (2.8)	21,474.2 (2.7)	17,635.0 (2.6)
COPD Bronchiectasis	114,691.0 (13.2)	81,693.0 (11.3)	98,302.4 (12.2)	77,114.6 (11.5)
Cirrhosis	13,133.0 (1.5)	13,347.0 (1.8)	12,123.6 (1.5)	10,261.1 (1.5)
Dementia	18,086.0 (2.1)	14,003.0 (1.9)	16,292.8 (2.0)	13,379.1 (2.0)
Immunocompromised[d]	64,316.0 (7.4)	55,795.0 (7.7)	57,012.5 (7.1)	46,773.2 (7.0)
Spinal cord injury	4,474.0 (0.5)	4,480.0 (0.6)	4,156.5 (0.5)	3,510.7 (0.5)
No CKD[e]	641,429.0 (73.9)	552,411.0 (76.2)	602,813.0 (74.7)	504,255.8 (75.1)
CKD[e]	199,994.0 (23.0)	155,027.0 (21.4)	182,042.9 (22.6)	149,474.2 (22.3)

	mRNA-1273 x3 Unweighted	BNT162b2 x3 Unweighted	mRNA-1273 x3 Weighted	BNT162b2 x3 Weighted
Severe CKD[f]	26,242.0 (3.0)	17,601.0 (2.4)	21,999.2 (2.7)	17,816.5 (2.7)
Dialysis	6,910.0 (0.8)	5,837.0 (0.8)	6,124.6 (0.8)	5,002.7 (0.7)
Cancer[g] solid organ	31,527.0 (3.6)	27,645.0 (3.8)	28,276.7 (3.5)	23,241.7 (3.5)
Cancer[g] lymphoma leukemia	11,804.0 (1.4)	9,299.0 (1.3)	10,418.8 (1.3)	8,369.0 (1.2)
Cancer[g] other	5,382.0 (0.6)	5,287.0 (0.7)	4,872.2 (0.6)	4,206.3 (0.6)
Home based primary care	16,240.0 (1.9)	10,287.0 (1.4)	13,054.2 (1.6)	9,583.1 (1.4)
Current smoker, n (%)	190,127.0 (21.9)	162,216.0 (22.4)	172,323.4 (21.4)	141,284.7 (21.0)
Alcohol use disorder[h], n (%)	58,261.0 (6.7)	53,246.0 (7.3)	52,786.8 (6.5)	43,738.4 (6.5)
Substance use disorder[i], n (%)	35,484.0 (4.1)	34,440.0 (4.8)	31,463.0 (3.9)	26,377.8 (3.9)
Housing problems[j], n (%)	28,965.0 (3.3)	28,549.0 (3.9)	25,619.6 (3.2)	21,587.8 (3.2)
CAN[k], n (%)				
0-49.9	248,046.0 (28.6)	236,698.0 (32.6)	240,281.8 (29.8)	204,831.6 (30.5)
50-74.9	287,883.0 (33.2)	234,097.0 (32.3)	266,185.7 (33.0)	220,566.6 (32.8)
75-100	331,736.0 (38.2)	254,244.0 (35.1)	300,387.6 (37.2)	246,148.3 (36.7)
Primary quarter, n (%)				
12/2020-02/2021	606,500.0 (69.9)	474,963.0 (65.5)	557,606.1 (69.1)	459,444.3 (68.4)
03/2021-05/2021	251,519.0 (29.0)	237,551.0 (32.8)	238,207.4 (29.5)	201,718.4 (30.0)
06/2021-08/2021	8,804.0 (1.0)	11,102.0 (1.5)	10,022.7 (1.2)	9,341.0 (1.4)
09/2021-11/2021	839.0 (0.1)	1,418.0 (0.2)	1,014.9 (0.1)	1,039.8 (0.2)
12/2021-02/2022	3.0 (0.0)	5.0 (0.0)	4.0 (0.0)	3.0 (0.0)

[a]Race/ethnicity was assessed using self-identified data found in Veteran Health Records.
[b]Urban/rural was assessed using defined based on the Rural Urban Commuting Area (RUCA) categories developed by the Department of Agriculture and Health and Human Services' Health Resource and Services Administration.
[c]91 or more days prior to boosted shot
[d]Immunocompromised definition based on medications and history of cancer (see supplement for list of meds).
[e]CKD defined as having a glomerular filtration rate between 30 and 60.
[f]Severe CKD defined as having a glomerular filtration rate <30.
[g]Cancer definition based on diagnosis codes. 2 outpatient or 1 inpatient diagnosis code in the VHA (see supplement).
[h]Alcohol use disorder defined as 1 outpatient or 1 inpatient code within 2 years of index.
[i]Including cannabis, opioids, inhalants.
[j]Housing problems defined as homelessness, inadequate housing, other problems related to housing and economic circumstances.
[k]The Care Assessment Need (CAN) Score is a predictive analytic tool that estimates the relative probability of hospitalization and death within 90 days or one-year from the calculation date. The oOffice of Clinical Systems Development and Evaluation (CSDE-10E2A) produces the weekly CAN Score Report to help identify the highest risk patients in a primary care panel or cohort. We used the one-year score.

Abbreviations: BMI: body mass index; CHF: chronic heart failure; CKD: chronic kidney disease; COPD: chronic obstructive pulmonary disease; TIA: transient ischemic attack

Unweighted samples: Standardized differences were less than 0.10 for all variables.
Weighted samples: Standardized differences were less than 0.04 for all variables.

Supplementary Table 7: Propensity weighted samples of veterans age ≥65 but no high-risk co-morbid conditions who have received three doses of mRNA-1273 or BNT-162b2 COVID-19 vaccines.

	mRNA-1273 x3 Unweighted	BNT162b2 x3 Unweighted	mRNA-1273 x3 Weighted	BNT162b2 x3 Weighted
N	118,313	96,864	111,286	91,051
Male, n (%)	113,000.0 (95.5)	92,217.0 (95.2)	106,221.40 (95.4)	86,929.7 (95.5)
Female, n (%)	5,313.0 (4.5)	4,647.0 (4.8)	5,064.40 (4.6)	4,121.7 (4.5)
Age, n (%)	76.2 (7.0)	75.7 (6.8)	76.10 (7.0)	76.0 (6.9)
Race[a], n (%)				
White	95,300.0 (80.5)	73,046.0 (75.4)	88,367.1 (79.4)	71,363.0 (78.4)
Black or African American	10,864.0 (9.2)	13,384.0 (13.8)	11,109.6 (10.0)	9,899.8 (10.9)
Other or unknown race	12,149.0 (10.3)	10,434.0 (10.8)	11,809.1 (10.6)	9,788.5 (10.8)
Hispanic or Latino Ethnicity (regardless of race), n (%)	7,370.0 (6.2)	5,251.0 (5.4)	6,433.9 (5.8)	5,167.3 (5.7)
Currently Married, n (%)	79,740.0 (67.4)	63,939.0 (66.0)	76,057.3 (68.3)	62,432.7 (68.6)
Highly Rural or unknown, n (%)	6,708.0 (5.7)	2,743.0 (2.8)	5,295.1 (4.8)	3,984.6 (4.4)
Rural, n (%)	39,184.0 (33.1)	22,177.0 (22.9)	34,557.1 (31.1)	25,935.3 (28.5)
Urban[b], n (%)	72,421.0 (61.2)	71,944.0 (74.3)	71,433.6 (64.2)	61,131.4 (67.1)
VHA Priority, n (%)				
1-4	61,256.0 (51.8)	52,202.0 (53.9)	58,468.0 (52.5)	48,151.8 (52.9)
5-6	27,306.0 (23.1)	21,185.0 (21.9)	24,965.5 (22.4)	20,128.0 (22.1)
7-8	29,751.0 (25.1)	23,477.0 (24.2)	27,852.3 (25.0)	22,771.6 (25)
Reinfection[c], n (%)	2,861.0 (2.4)	2,657.0 (2.7)	2,645.5 (2.4)	2,224.6 (2.4)
BMI, n (%)				
<18.5	738.0 (0.6)	667.0 (0.7)	710.7 (0.6)	589.6 (0.6)
18.5-24.9	32,677.0 (27.6)	26,975.0 (27.8)	30,656.8 (27.5)	25,065.4 (27.5)
25-29.9	69,154.0 (58.5)	57,654.0 (59.5)	65,387.5 (58.8)	53,648.5 (58.9)
BMI unknown	15,744.0 (13.3)	11,568.0 (11.9)	14,530.8 (13.1)	11,747.9 (12.9)
Comorbidities associated with severe COVID-19 illness, n (%)				
Hypertension	55,668.0 (47.1)	43,720.0 (45.1)	51,132.5 (45.9)	41,397.0 (45.5)
Spinal cord injury	598.0 (0.5)	550.0 (0.6)	543.0 (0.5)	435.5 (0.5)
Dialysis	51.0 (0.0)	45.0 (0.0)	53.4 (0.0)	43.0 (0.0)
Home based primary care	1,038.0 (0.9)	682.0 (0.7)	874.3 (0.8)	643.4 (0.7)
Alcohol use disorder[d], n (%)	4,443.0 (3.8)	3,738.0 (3.9)	4,061.9 (3.7)	3,279.0 (3.6)
Substance use disorder[e], n (%)	1,742.0 (1.5)	1,564.0 (1.6)	1,552.5 (1.4)	1,257.5 (1.4)
Housing problems[f], n (%)	1,497.0 (1.3)	1,405.0 (1.5)	1,334.1 (1.2)	1,097.7 (1.2)
CAN[g], n (%)				
0-49.9	28,368.0 (24.0)	23,442.0 (24.2)	26,640.6 (23.9)	21,709.0 (23.8)
50-74.9	48,845.0 (41.3)	40,093.0 (41.4)	45,795.4 (41.2)	37,459.8 (41.1)
75-100	41,100.0 (34.7)	33,329.0 (34.4)	38,849.8 (34.9)	31,882.7 (35.0)
Primary quarter, n (%)				
12/2020-02/2021	95,160.0 (80.4)	76,385.0 (78.9)	88,782.3 (79.8)	72,325.8 (79.4)

	mRNA-1273 x3 Unweighted	BNT162b2 x3 Unweighted	mRNA-1273 x3 Weighted	BNT162b2 x3 Weighted
03/2021-05/2021	22,445.0 (19.0)	19,532.0 (20.2)	21,653.6 (19.5)	17,942.5 (19.7)
06/2021-08/2021	653.0 (0.6)	854.0 (0.9)	775.8 (0.7)	710.0 (0.8)
09/2021-11/2021	55.0 (0.0)	93.0 (0.1)	74.2 (0.1)	73.1 (0.1)

[a]Race/ethnicity was assessed using self-identified data found in Veteran Health Records.
[b]Urban/rural was assessed using defined based on the Rural Urban Commuting Area (RUCA) categories developed by the Department of Agriculture and Health and Human Services' Health Resource and Services Administration.
[c]91 or more days prior to boosted shot
[d]Alcohol use disorder defined as 1 outpatient or 1 inpatient code within 2 years of index.
[e]Including cannabis, opioids, inhalants.
[f]Housing problems defined as homelessness, inadequate housing, other problems related to housing and economic circumstances.
[g]The Care Assessment Need (CAN) Score is a predictive analytic tool that estimates the relative probability of hospitalization and death within 90 days or one-year from the calculation date. The oOffice of Clinical Systems Development and Evaluation (CSDE-10E2A) produces the weekly CAN Score Report to help identify the highest risk patients in a primary care panel or cohort. We used the one-year score.

Abbreviations: BMI: body mass index; CHF: chronic heart failure; CKD: chronic kidney disease; COPD: chronic obstructive pulmonary disease; TIA: transient ischemic attack

Unweighted samples: Standardized differences were less than 0.13 for all variables.
Weighted samples: Standardized differences were less than 0.03 for all variables.

Supplementary Table 8: Propensity weighted samples of veterans with high-risk co-morbid conditions but not immunocompromised who have received three doses of mRNA-1273 or BNT-162b2 COVID-19 vaccines.

	mRNA-1273 x3 Unweighted	BNT162b2 x3 Unweighted	mRNA-1273 x3 Weighted	BNT162b2 x3 Weighted
N	647,603	540,867	602,356	500,452
Male, n (%)	605,917.0 (93.6)	497,561.0 (92.0)	560,642.3 (93.1)	464,558.1 (92.8)
Female, n (%)	41,686.0 (6.4)	43,306.0 (8.0)	41,713.7 (6.9)	35,894.3 (7.2)
Age, n (%)	70.2 (11.9)	68.3 (12.6)	69.8 (12.2)	69.5 (12.38)
Race[a], n (%)				
White	476,532.0 (73.6)	355,921.0 (65.8)	433,515.7 (72.0)	352,564.5 (70.4)
Black or African American	109,603.0 (16.9)	129,779.0 (24.0)	109,203.4 (18.1)	97,520.9 (19.5)
Unknown	61,468.0 (9.5)	55,167.0 (10.2)	59,636.9 (9.9)	50,367.0 (10.1)
Hispanic or Latino Ethnicity (regardless of race), n (%)	46,196.0 (7.1)	36,460.0 (6.7)	41,618.7 (6.9)	34,211.7 (6.8)
Currently Married, n (%)	395,600.0 (61.1)	316,317.0 (58.5)	372,918.7 (61.9)	310,570.2 (62.1)
Highly Rural or unknown, n (%)	32,809.0 (5.1)	12,803.0 (2.4)	25,793.2 (4.3)	19,232.7 (3.8)
Rural, n (%)	216,739.0 (33.5)	118,593.0 (21.9)	186,919.3 (31.0)	139,880.1 (28)
Urban[b], n (%)	398,055.0 (61.5)	409,471.0 (75.7)	389,643.6 (64.7)	341,339.6 (68.2)
VHA Priority, n (%)				
1-4	410,693.0 (63.4)	353,169.0 (65.3)	385,721.8 (64.0)	321,925.1 (64.3)
5-6	124,093.0 (19.2)	97,694.0 (18.1)	110,944.5 (18.4)	90,114.8 (18)
7-8	112,817.0 (17.4)	90,004.0 (16.6)	105,689.7 (17.5)	88,412.5 (17.7)
Reinfection[c], n (%)	30,093.0 (4.6)	28,825.0 (5.3)	27,617.3 (4.6)	23,592.0 (4.7)
BMI, n (%)				
<18.5	4,102.0 (0.6)	3,322.0 (0.6)	3,646.0 (0.6)	2,951.8 (0.6)
18.5-24.9	87,770.0 (13.6)	70,429.0 (13.0)	79,512.7 (13.2)	65,274.9 (13)
25-29.9	180,519.0 (27.9)	146,559.0 (27.1)	166,215.0 (27.6)	137,672.3 (27.5)
>=30	343,407.0 (53.0)	300,109.0 (55.5)	324,756.6 (53.9)	272,008.9 (54.4)
BMI unknown	31,805.0 (4.9)	20,448.0 (3.8)	28,225.8 (4.7)	22,544.6 (4.5)
Comorbidities associated with severe COVID-19 illness, n (%)				
Hypertension	438,778.0 (67.8)	348,853.0 (64.5)	398,520.7 (66.2)	326,324.9 (65.2)
CHF	43,323.0 (6.7)	33,971.0 (6.3)	37,620.1 (6.2)	30,121.9 (6)
IHD	142,018.0 (21.9)	106,488.0 (19.7)	127,674.6 (21.2)	103,398.5 (20.7)
Diabetes	271,132.0 (41.9)	215,731.0 (39.9)	245,916.8 (40.8)	201,142.0 (40.2)
Stroke TIA	18,261.0 (2.8)	15,538.0 (2.9)	16,341.6 (2.7)	13,356.1 (2.7)
COPD Bronchiectasis	90,362.0 (14.0)	63,218.0 (11.7)	77,955.5 (12.9)	61,105.9 (12.2)
Cirrhosis	9,758.0 (1.5)	9,695.0 (1.8)	9,002.9 (1.5)	7,561.2 (1.5)
Dementia	15,853.0 (2.4)	12,229.0 (2.3)	14,369.8 (2.4)	11,809.5 (2.4)
Spinal cord injury	3,080.0 (0.5)	3,111.0 (0.6)	2,889.9 (0.5)	2,445.4 (0.5)
No CKD[d]	451,796.0 (69.8)	392,894.0 (72.6)	424,886.2 (70.5)	354,769.5 (70.9)
CKD[d]	173,618.0 (26.8)	133,674.0 (24.7)	158,912.0 (26.4)	130,710.2 (26.1)
Severe CKD[e]	22,189.0 (3.4)	14,299.0 (2.6)	18,557.8 (3.1)	14,972.6 (3)

	mRNA-1273 x3 Unweighted	BNT162b2 x3 Unweighted	mRNA-1273 x3 Weighted	BNT162b2 x3 Weighted
Dialysis	5,074.0 (0.8)	4,237.0 (0.8)	4,523.2 (0.8)	3,679.4 (0.7)
Home based primary care	12,217.0 (1.9)	7,737.0 (1.4)	9,880.0 (1.6)	7,293.5 (1.5)
Current smoker, n (%)	166,203.0 (25.7)	141,415.0 (26.1)	151,414.4 (25.1)	124,124.1 (24.8)
Alcohol use disorder[f], n (%)	45,641.0 (7.0)	41,583.0 (7.7)	41,357.1 (6.9)	34,175.4 (6.8)
Substance use disorder[g], n (%)	27,783.0 (4.3)	26,770.0 (4.9)	24,599.2 (4.1)	20,506.2 (4.1)
Housing problems[h], n (%)	22,894.0 (3.5)	22,497.0 (4.2)	20,261.1 (3.4)	17,023.5 (3.4)
CAN[i], n (%)				
0-49.9	199,353.0 (30.8)	192,894.0 (35.7)	193,779.6 (32.2)	165,231.3 (33.0)
50-74.9	212,693.0 (32.8)	171,403.0 (31.7)	195,828.7 (32.5)	161,523.0 (32.3)
75-100	235,557.0 (36.4)	176,570.0 (32.6)	212,747.7 (35.3)	173,698.1 (34.7)
Primary quarter, n (%)				
12/2020-02/2021	438,020.0 (67.6)	339,626.0 (62.8)	402,586.8 (66.8)	330,817.8 (66.1)
03/2021-05/2021	201,834.0 (31.2)	191,303.0 (35.4)	190,912.7 (31.7)	161,343.1 (32.2)
06/2021-08/2021	7,110.0 (1.1)	8,822.0 (1.6)	8,078.4 (1.3)	7,491.0 (1.5)
09/2021-11/2021	639.0 (0.1)	1,116.0 (0.2)	778.1 (0.1)	800.6 (0.2)

[a]Race/ethnicity was assessed using self-identified data found in Veteran Health Records.
[b]Urban/rural was assessed using defined based on the Rural Urban Commuting Area (RUCA) categories developed by the Department of Agriculture and Health and Human Services' Health Resource and Services Administration.
[c]91 or more days prior to boosted shot
[d]CKD defined as having a glomerular filtration rate between 30 and 60.
[e]Severe CKD defined as having a glomerular filtration rate <30.
[f]Alcohol use disorder defined as 1 outpatient or 1 inpatient code within 2 years of index.
[g]Including cannabis, opioids, inhalants.
[h]Housing problems defined as homelessness, inadequate housing, other problems related to housing and economic circumstances.
[i]The Care Assessment Need (CAN) Score is a predictive analytic tool that estimates the relative probability of hospitalization and death within 90 days or one-year from the calculation date. The oOffice of Clinical Systems Development and Evaluation (CSDE-10E2A) produces the weekly CAN Score Report to help identify the highest risk patients in a primary care panel or cohort. We used the one-year score.

Abbreviations: BMI: body mass index; CHF: chronic heart failure; CKD: chronic kidney disease; COPD: chronic obstructive pulmonary disease; TIA: transient ischemic attack

Unweighted samples: Standardized differences were less than 0.14 for all variables.
Weighted samples: Standardized differences were less than 0.04 for all variables.

Supplementary Table 9: Propensity weighted samples of veterans with an immunocompromising condition who have received three doses of mRNA-1273 or BNT-162b2 COVID-19 vaccines.

	mRNA-1273 x3 Unweighted	BNT162b2 x3 Unweighted	mRNA-1273 x3 Weighted	BNT162b2 x3 Weighted
N	101,749	87,308	90,912	76,237
Male, n (%)	94,613.0 (93.0)	79,859.0 (91.5)	84,040.3 (92.4)	70,221.8 (92.1)
Female, n (%)	7,136.0 (7.0)	7,449.0 (8.5)	6,871.3 (7.6)	6,015.1 (7.9)
Age[a], n (%)	70.3 (10.7)	68.7 (11.4)	69.9 (11.0)	69.5 (11.3)
Race, n (%)				
White	75,987.0 (74.7)	57,550.0 (65.9)	66,491.7 (73.1)	53,837.5 (70.6)
Black or African American	17,495.0 (17.2)	22,269.0 (25.5)	16,724.8 (18.4)	15,988.2 (21.0)
Unknown	8,267.0 (8.1)	7,489.0 (8.6)	7,695.0 (8.5)	6,411.2 (8.4)
Hispanic or Latino Ethnicity (regardless of race), n (%)	6,804.0 (6.7)	5,555.0 (6.4)	5,986.4 (6.6)	5,057.7 (6.6)
Currently Married, n (%)	58,933.0 (57.9)	47,186.0 (54.0)	53,110.6 (58.4)	44,118.4 (57.9)
Highly Rural or unknown, n (%)	4,902.0 (4.8)	1,887.0 (2.2)	3,785.7 (4.2)	2,676.8 (3.5)
Rural, n (%)	32,983.0 (32.4)	18,231.0 (20.9)	27,417.5 (30.2)	19,704.3 (25.8)
Urban[b], n (%)	63,864.0 (62.8)	67,190.0 (77.0)	59,708.4 (65.7)	53,855.8 (70.6)
VHA Priority, n (%)				
1-4	67,010.0 (65.9)	58,190.0 (66.6)	60,258.0 (66.3)	50,726.8 (66.5)
5-6	20,696.0 (20.3)	17,514.0 (20.1)	17,873.5 (19.7)	14,867.6 (19.5)
7-8	14,043.0 (13.8)	11,604.0 (13.3)	12,780.1 (14.1)	10,642.5 (14.0)
Reinfection[c], n (%)	6,767.0 (6.7)	6,858.0 (7.9)	6,119.9 (6.7)	5,429.5 (7.1)
BMI, n (%)				
<18.5	1,205.0 (1.2)	1,119.0 (1.3)	1,049.9 (1.2)	894.2 (1.2)
18.5-24.9	19,933.0 (19.6)	17,338.0 (19.9)	17,665.0 (19.4)	14,898.1 (19.5)
25-29.9	35,037.0 (34.4)	30,407.0 (34.8)	31,480.9 (34.6)	26,688.9 (35.0)
>=30	41,785.0 (41.1)	35,951.0 (41.2)	37,425.1 (41.2)	31,121.2 (40.8)
BMI unknown	3,789.0 (3.7)	2,493.0 (2.9)	3,290.8 (3.6)	2,634.5 (3.5)
Comorbidities associated with severe COVID-19 illness, n (%)				
Hypertension	72,014.0 (70.8)	59,694.0 (68.4)	63,312.7 (69.6)	52,370.8 (68.7)
CHF	10,317.0 (10.1)	8,577.0 (9.8)	8,715.7 (9.6)	7,114.1 (9.3)
IHD	24,059.0 (23.6)	18,857.0 (21.6)	20,796.5 (22.9)	16,936.7 (22.2)
Diabetes	37,157.0 (36.5)	30,520.0 (35.0)	32,353.0 (35.6)	26,687.4 (35.0)
Stroke TIA	3,764.0 (3.7)	3,365.0 (3.9)	3,276.3 (3.6)	2,760.0 (3.6)
COPD Bronchiectasis	24,329.0 (23.9)	18,475.0 (21.2)	20,337.5 (22.4)	16,275.6 (21.3)
Cirrhosis	3,375.0 (3.3)	3,652.0 (4.2)	3,118.7 (3.4)	2,746.0 (3.6)
Dementia	2,233.0 (2.2)	1,774.0 (2.0)	1,927.3 (2.1)	1,617.4 (2.1)
Immunocompromised[d]	64,316.0 (63.2)	55,795.0 (63.9)	57,173.5 (62.9)	48,101.2 (63.1)
Spinal cord injury	796.0 (0.8)	819.0 (0.9)	718.8 (0.8)	633.4 (0.8)
No CKD[e]	71,320.0 (70.1)	62,653.0 (71.8)	64,253.9 (70.7)	54,137.1 (71.0)
CKD[e]	26,376.0 (25.9)	21,353.0 (24.5)	23,217.8 (25.5)	19,207.9 (25.2)

	mRNA-1273 x3 Unweighted	BNT162b2 x3 Unweighted	mRNA-1273 x3 Weighted	BNT162b2 x3 Weighted
Severe CKD[f]	4,053.0 (4.0)	3,302.0 (3.8)	3,439.8 (3.8)	2,891.9 (3.8)
Dialysis	1,785.0 (1.8)	1,555.0 (1.8)	1,541.1 (1.7)	1,309.1 (1.7)
Cancer[g] solid organ	31,527.0 (31.0)	27,645.0 (31.7)	28,402.2 (31.2)	23,901.1 (31.4)
Cancer[g] lymphoma leukemia	11,804.0 (11.6)	9,299.0 (10.7)	10,500.4 (11.6)	8,626.0 (11.3)
Cancer[g] other	3,739.0 (3.7)	3,751.0 (4.3)	3,390.2 (3.7)	2,953.9 (3.9)
Home based primary care	2,985.0 (2.9)	1,868.0 (2.1)	2,286.3 (2.5)	1,651.9 (2.2)
Current smoker, n (%)	23,924.0 (23.5)	20,801.0 (23.8)	20,884.9 (23)	17,277.6 (22.7)
Alcohol use disorder[h], n (%)	8,177.0 (8.0)	7,925.0 (9.1)	7,290.0 (8.0)	6,187.5 (8.1)
Substance use disorder[i], n (%)	5,959.0 (5.9)	6,106.0 (7.0)	5,265.2 (5.8)	4,616.8 (6.1)
Housing problems[j], n (%)	4,574.0 (4.5)	4,647.0 (5.3)	3,974.5 (4.4)	3,472.1 (4.6)
CAN[k], n (%)				
0-49.9	20,325.0 (20.0)	20,362.0 (23.3)	19,276.5 (21.2)	16,886.8 (22.2)
50-74.9	26,345.0 (25.9)	22,601.0 (25.9)	23,624.7 (26.0)	19,814.0 (26.0)
75-100	55,079.0 (54.1)	44,345.0 (50.8)	48,010.4 (52.8)	39,536.0 (51.9)
Primary quarter, n (%)				
12/2020-02/2021	73,320.0 (72.1)	58,952.0 (67.5)	64,594.7 (71.1)	53,100.7 (69.7)
03/2021-05/2021	27,240.0 (26.8)	26,716.0 (30.6)	25,000.4 (27.5)	21,825.2 (28.6)
06/2021-08/2021	1,041.0 (1.0)	1,426.0 (1.6)	1,155.3 (1.3)	1,143.0 (1.5)
09/2021-11/2021	145.0 (0.1)	209.0 (0.2)	156.8 (0.2)	164.9 (0.2)

[a]Race/ethnicity was assessed using self-identified data found in Veteran Health Records.
[b]Urban/rural was assessed using defined based on the Rural Urban Commuting Area (RUCA) categories developed by the Department of Agriculture and Health and Human Services' Health Resource and Services Administration.
[c]91 or more days prior to boosted shot
[d]Immunocompromised definition based on medications and history of cancer (see supplement for list of meds).
[e]CKD defined as having a glomerular filtration rate between 30 and 60.
[f]Severe CKD defined as having a glomerular filtration rate <30.
[g]Cancer definition based on diagnosis codes. 2 outpatient or 1 inpatient diagnosis code in the VHA (see supplement).
[h]Alcohol use disorder defined as 1 outpatient or 1 inpatient code within 2 years of index.
[i]Including cannabis, opioids, inhalants.
[j]Housing problems defined as homelessness, inadequate housing, other problems related to housing and economic circumstances.
[k]The Care Assessment Need (CAN) Score is a predictive analytic tool that estimates the relative probability of hospitalization and death within 90 days or one-year from the calculation date. The oOffice of Clinical Systems Development and Evaluation (CSDE-10E2A) produces the weekly CAN Score Report to help identify the highest risk patients in a primary care panel or cohort. We used the one-year score.

Abbreviations: BMI: body mass index; CHF: chronic heart failure; CKD: chronic kidney disease; COPD: chronic obstructive pulmonary disease; TIA: transient ischemic attack

Unweighted samples: Standardized differences were less than 0.14 for all variables.
Weighted samples: Standardized differences were less than 0.05 for all variables.
