# Supplementary material for: Comparative mRNA booster effectiveness against death or hospitalization with COVID-19 pneumonia across at-risk US Veteran populations
Source: Nat Commun. 2023 May 23;14:2976. doi: 10.1038/s41467-023-38503-8 (PMC10205032; doi:10.1038/s41467-023-38503-8)
Supplement: Supplementary file 2 — Reporting Summary [file 41467_2023_38503_MOESM2_ESM.pdf]

nature portfolio

Corresponding author(s): J. Daniel Kelly

Last updated by author(s): May 4, 2023

Reporting Summary

Nature Portfolio wishes to improve the reproducibility of the work that we publish. This form provides structure for consistency and transparency in reporting. For further information on Nature Portfolio policies, see our Editorial Policies and the Editorial Policy Checklist.

Statistics

For all statistical analyses, confirm that the following items are present in the figure legend, table legend, main text, or Methods section.

n/a	Confirmed	
☐	☒	The exact sample size (*n*) for each experimental group/condition, given as a discrete number and unit of measurement
☐	☒	A statement on whether measurements were taken from distinct samples or whether the same sample was measured repeatedly
☐	☒	The statistical test(s) used AND whether they are one- or two-sided<br>*Only common tests should be described solely by name; describe more complex techniques in the Methods section.*
☐	☒	A description of all covariates tested
☐	☒	A description of any assumptions or corrections, such as tests of normality and adjustment for multiple comparisons
☐	☒	A full description of the statistical parameters including central tendency (e.g. means) or other basic estimates (e.g. regression coefficient) AND variation (e.g. standard deviation) or associated estimates of uncertainty (e.g. confidence intervals)
☐	☒	For null hypothesis testing, the test statistic (e.g. *F*, *t*, *r*) with confidence intervals, effect sizes, degrees of freedom and *P* value noted<br>*Give P values as exact values whenever suitable.*
☒	☐	For Bayesian analysis, information on the choice of priors and Markov chain Monte Carlo settings
☐	☒	For hierarchical and complex designs, identification of the appropriate level for tests and full reporting of outcomes
☒	☐	Estimates of effect sizes (e.g. Cohen's *d*, Pearson's *r*), indicating how they were calculated

Our web collection on statistics for biologists contains articles on many of the points above.

Software and code

Policy information about availability of computer code

Data collection	No software was used for data collection.
Data analysis	Data analysis was performed with RStudio 2022.07.02 build 576 running R version 4.1.2. The survival R package (Therneau 2023 v3.5-0) and rms R package (Harrell 2023 v6.4-0) were used to perform statistical analyses.

For manuscripts utilizing custom algorithms or software that are central to the research but not yet described in published literature, software must be made available to editors and reviewers. We strongly encourage code deposition in a community repository (e.g. GitHub). See the Nature Portfolio guidelines for submitting code & software for further information.

Data

Policy information about availability of data

All manuscripts must include a data availability statement. This statement should provide the following information, where applicable:

- Accession codes, unique identifiers, or web links for publicly available datasets
- A description of any restrictions on data availability
- For clinical datasets or third party data, please ensure that the statement adheres to our policy

Data to generate the findings of this study are available from the US Department of Veterans Affairs. VA data are made freely available to researchers behind the VA firewall through the VA Informatics and Computing Infrastructure (VINCI) and with an approved VA study protocol. More information is available at https://www.virec.research.va.gov. Source data are provided with this paper.

Human research participants

Policy information about studies involving human research participants and Sex and Gender in Research.

Reporting on sex and gender	Sex as assigned at birth was used for all analyses. All analytical models adjusted for sex. Gender-specific models/outcomes were not provided due to the rarity of the COVID-19 pneumonia outcome and relatively small population of female Veterans.
Population characteristics	Relevant population characteristics are:<br><br>Demographics: patient age, sex, race, Hispanic ethnicity, marital status, census-designated urban/rural status<br><br>Medical characteristics: vaccination/booster for COVID-19; history of COVID-19 infection; receipt of home based primary care; spinal cord injury; past diagnoses for hypertension, CHF, ischemic heart disease, diabetes, stroke/TIA, COPD, bronchiectasis, cirrhosis, cancer, dementia, housing insecurity, alcohol use, and other drug use; patient smoking status; receipt of immunocompromising medications; receipt of dialysis treatment; BMI; Care Assessment Needs (CAN) score (a measure of patient medical utilization within the VA)
Recruitment	No patient recruitment occurred for this study.
Ethics oversight	The institutional review board of the University of California, San Francisco approved this study and waived requirement for patient consent as it involved no more than minimal risk to patients.

Note that full information on the approval of the study protocol must also be provided in the manuscript.

Field-specific reporting

Please select the one below that is the best fit for your research. If you are not sure, read the appropriate sections before making your selection.

☒ Life sciences ☐ Behavioural & social sciences ☐ Ecological, evolutionary & environmental sciences

For a reference copy of the document with all sections, see nature.com/documents/nr-reporting-summary-flat.pdf

Life sciences study design

All studies must disclose on these points even when the disclosure is negative.

Sample size	No a priori sample size determination was made. All patients who received primary care at a Veterans Administration medical center and who received at least three doses of the Moderna or Pfizer COVID-19 vaccine/booster and who met pre-determined inclusion criteria (described below) were included for analysis.
Data exclusions	Exclusion rationale were to represent community-dwelling individuals and to avoid misclassification of individuals misdiagnosed with COVID-19.
Replication	Replication and reproducibility are important, so we have provided access in the Data and Code Availability Statements.
Randomization	Allocation to treatment arms was not random. Authors used propensity methods to adjust for the confounding effect of nonrandom treatment assignment.
Blinding	This was secondary data analysis in which use of blinding was not indicated.

Reporting for specific materials, systems and methods

We require information from authors about some types of materials, experimental systems and methods used in many studies. Here, indicate whether each material, system or method listed is relevant to your study. If you are not sure if a list item applies to your research, read the appropriate section before selecting a response.

Materials & experimental systems

n/a	Involved in the study
☒	☐ Antibodies
☒	☐ Eukaryotic cell lines
☒	☐ Palaeontology and archaeology
☒	☐ Animals and other organisms
☒	☐ Clinical data
☒	☐ Dual use research of concern

Methods

n/a	Involved in the study
☒	☐ ChIP-seq
☒	☐ Flow cytometry
☒	☐ MRI-based neuroimaging
